# Outer Hair Cell Lateral Wall Structure Constrains the Mobility of Plasma Membrane Proteins

**DOI:** 10.1371/journal.pgen.1005500

**Published:** 2015-09-09

**Authors:** Tetsuji Yamashita, Pierre Hakizimana, Siva Wu, Ahmed Hassan, Stefan Jacob, Jamshid Temirov, Jie Fang, Marcia Mellado-Lagarde, Richard Gursky, Linda Horner, Barbara Leibiger, Sara Leijon, Victoria E. Centonze, Per-Olof Berggren, Sharon Frase, Manfred Auer, William E. Brownell, Anders Fridberger, Jian Zuo

**Affiliations:** 1 Department of Developmental Neurobiology, St. Jude Children’s Research Hospital, Memphis, Tennessee, United States of America; 2 Department of Clinical and Experimental Medicine, Neuroscience, Linköping University, Linköping, Sweden; 3 Karolinska Institutet, Center for Hearing and Communication Research, Department of Clinical Science, Intervention, and Technology, M1, Karolinska University Hospital, Stockholm, Sweden; 4 Life Sciences Division, Lawrence Berkeley National Laboratory, Berkeley, California, United States of America; 5 The Rolf Luft Research Center for Diabetes and Endocrinology, Karolinska Institutet, Stockholm, Sweden; 6 Cell and Tissue Imaging Facility, St Jude Children’s Research Hospital, Memphis, Tennessee, United States of America; 7 School of Pharmacy and Biomolecular Sciences, University of Brighton, Brighton, United Kingdom; 8 Bobby R. Alford Department of Otolaryngology, Head & Neck Surgery, and Department of Neuroscience, Baylor College of Medicine, Houston, Texas, United States of America; Stanford University School of Medicine, UNITED STATES

## Abstract

Nature’s fastest motors are the cochlear outer hair cells (OHCs). These sensory cells use a membrane protein, Slc26a5 (prestin), to generate mechanical force at high frequencies, which is essential for explaining the exquisite hearing sensitivity of mammalian ears. Previous studies suggest that Slc26a5 continuously diffuses within the membrane, but how can a freely moving motor protein effectively convey forces critical for hearing? To provide direct evidence in OHCs for freely moving Slc26a5 molecules, we created a knockin mouse where Slc26a5 is fused with YFP. These mice and four other strains expressing fluorescently labeled membrane proteins were used to examine their lateral diffusion in the OHC lateral wall. All five proteins showed minimal diffusion, but did move after pharmacological disruption of membrane-associated structures with a cholesterol-depleting agent and salicylate. Thus, our results demonstrate that OHC lateral wall structure constrains the mobility of plasma membrane proteins and that the integrity of such membrane-associated structures are critical for Slc26a5’s active and structural roles. The structural constraint of membrane proteins may exemplify convergent evolution of cellular motors across species. Our findings also suggest a possible mechanism for disorders of cholesterol metabolism with hearing loss such as Niemann-Pick Type C diseases.

## Author Summary

Nature’s fastest motor is the cochlear outer hair cell (OHC) in the mammalian inner ear. These cells can contract and elongate thousands of times per second. Slc26a5 (prestin) is the essential protein in the fast motor and resides in the plasma membrane of OHC lateral wall. Slc26a5 undergoes voltage-dependent conformational changes associated with the rapid changes in cell length to increase mammalian hearing sensitivity. However, it remains unclear how Slc26a5 transfers the force created to the entire cell. In this study, we show the importance of association between Slc26a5 and specialized membrane structures of the OHC lateral wall. Mobility of Slc26a5 was normally constrained in membrane-associated structures and disruption of these structures by a cholesterol depleting reagent and salicylate liberated Slc26a5 and four other heterologously expressed membrane proteins. These observations provide evidence that OHC lateral wall structure constrains the mobility of plasma membrane proteins and such membrane-associated structures are critical for Slc26a5’s functional roles. Our findings also shed light on other cellular motors across species and suggest a mechanism for cholesterol metabolic disorders in humans.

## Introduction

The cylindrical cochlear sensory outer hair cells (OHCs) in the inner ear convert membrane potential changes into mechanical force at high frequencies [1, 2]. This force production greatly boosts the sound-evoked displacements of the hearing organ and is therefore required for establishing the remarkable sensitivity and frequency resolution of mammalian hearing organs [2–8].

**Fig 1:**
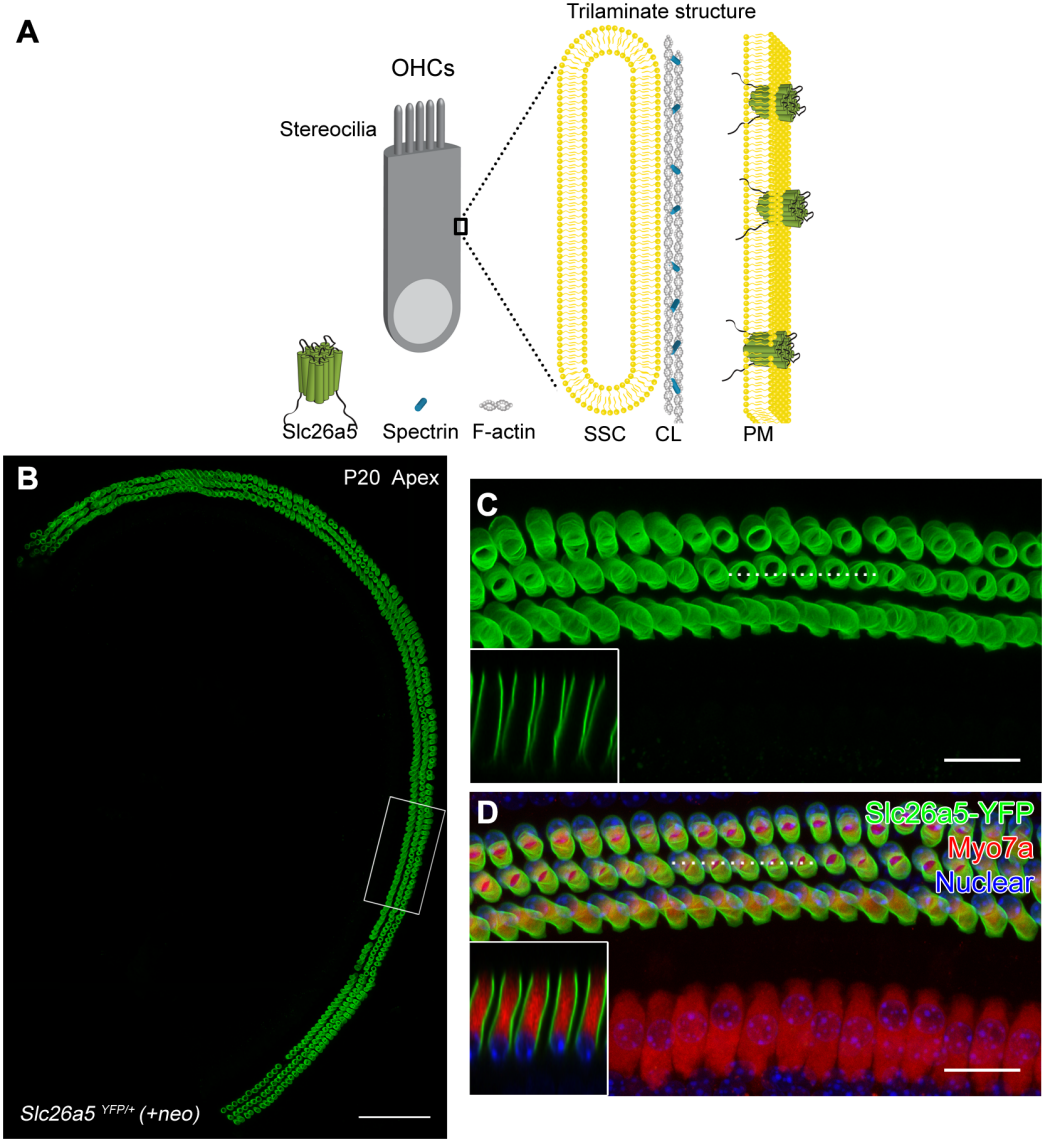
Slc26a5-YFP recapitulates endogenous Slc26a5 distribution and is functional in *Slc26a5* ^*YFP/+*^ *(+neo)* mice. (A) Schematic illustration shows morphology of OHCs and the OHC lateral wall. The lateral wall of OHCs consists of a unique trilaminate structure composed of PM, actin-spectrin cortical lattice (CL), and SSC. A more detailed model is shown in S9 Fig. (B-D) Slc26a5-YFP fluorescence (green) in the apical turn of the cochleae from *Slc26a5* ^*YFP/+*^ *(+neo)* mice at P20. White square in B is enlarged in C. The dashed line in C indicates the position of the optical xz-plane shown in the inset. Myo7a (red) is labeled as a HC marker in D. Counter-staining of nuclei (blue) using DAPI is shown in D. The dashed line in D indicates the position of the optical xz-plane shown in the inset.

OHC force production involves conformational changes of Slc26a5 (prestin), which is highly enriched in the cell’s lateral wall [9]. The OHC lateral wall contains a network of actin and spectrin filaments as well as endoplasmic reticulum immediately (Fig 1A) abutting the plasma membrane (PM), forming a trilaminate organization [10]. Several previous studies suggested that Slc26a5 is freely mobile and continuously diffusing [11–13]. However, how can a freely moving Slc26a5 transfer force to the cell to have such a profound effect on hearing?

To provide direct evidence of Slc26a5 mobility in OHCs, we created a novel knockin mouse where OHCs expressed a fusion protein of Slc26a5 and monomeric Venus yellow fluorescent protein (Slc26a5-YFP). Slc26a5-YFP function was indistinguishable from wild-type Slc26a5 and the distribution recapitulated endogenous Slc26a5 both during development and in mature cells. Strikingly, there was no diffusion of Slc26a5 either *in situ* or in isolated OHCs. Four other fluorescent proteins expressed in the OHC lateral wall also showed minimal lateral mobility, but pharmacological treatments that collapsed trilaminate structures in the OHC lateral wall liberated all tested fluorescently labeled membrane proteins, which began to diffuse. These results show that OHC lateral wall structure constrains the mobility of plasma membrane proteins and are critical for Slc26a5’s active and structural roles. Our findings also have implications in convergence evolution of cellular motors and in some disorders of cholesterol metabolism.

## Results

### Creation and characterization of the novel Slc26a5-YFP knockin mice

To specifically label Slc26a5 *in vivo*, it was fused with the Venus yellow fluorescent protein (YFP), which is brighter than traditional green-fluorescent proteins and insensitive to both pH and Cl^-^ [14]. To avoid artificial oligomerization, monomeric venus YFP (A206K) was used. A construct where YFP was fused at Slc26a5’s C-terminus was found to have robust nonlinear capacitance, a hallmark of Slc26a5 activity, when expressed in 293T cells (S1A Fig). In contrast, fusion of YFP at the N-terminus of Slc26a5 resulted in loss of nonlinear capacitance (S1A Fig). We therefore created a knockin construct where YFP was inserted right before the Slc26a5 termination codon, followed by a loxP-flanked neo cassette for embryonic stem cell screening (S1B Fig). The presence of YFP at the C-terminus of Slc26a5 in germline-transmitted mice was confirmed by Southern blot and PCR analysis (S1C and S1D Fig).

### The distribution of Slc26a5-YFP is normal in both adult and developing cochleae

At postnatal day 20 (P20) and later stages, YFP fluorescence signals were present in the lateral wall of *Slc26a5* ^*YFP/+*^ *(+neo)* OHCs, but absent from the top and bottom of the cells (Fig 1B–1D; n = 5). YFP was observed in OHCs in all cochlear turns but not in other regions of the inner ear (Fig 1B). To confirm that YFP-positive cells were indeed OHCs, we co-labeled them with Myo7a antibodies, and found that YFP fluorescence only was present in Myo7a-positive cells (Fig 1D). These results are consistent with the normal distribution of Slc26a5 [15].

Localization of the Slc26a5-YFP fluorescence was also investigated during postnatal development. At P5, Slc26a5-YFP fluorescence was observed in the cytosol (S2A–S2D Fig) near the stereociliary pole of OHCs. The expression appeared stronger in cells near the base of the cochlea; labeling with antibodies to the hair cell marker Myo6 (S2C and S2D Fig) confirmed that the cells were OHCs. These findings, which are consistent with previous reports [16, 17], confirm that Slc26a5-YFP recapitulates the endogenous Slc26a5 distribution in developing cochleae.

Interestingly, YFP fluorescence was not observed in the vestibular system or sperm (S2E–S2H Fig). Similar distribution of Slc26a5-YFP was observed in *Slc26a5* ^*YFP/YFP*^ *(+neo)* mice in developing and adult cochleae (S3 Fig).

### Slc26a5-YFP is functional in OHCs

**Fig 2:**
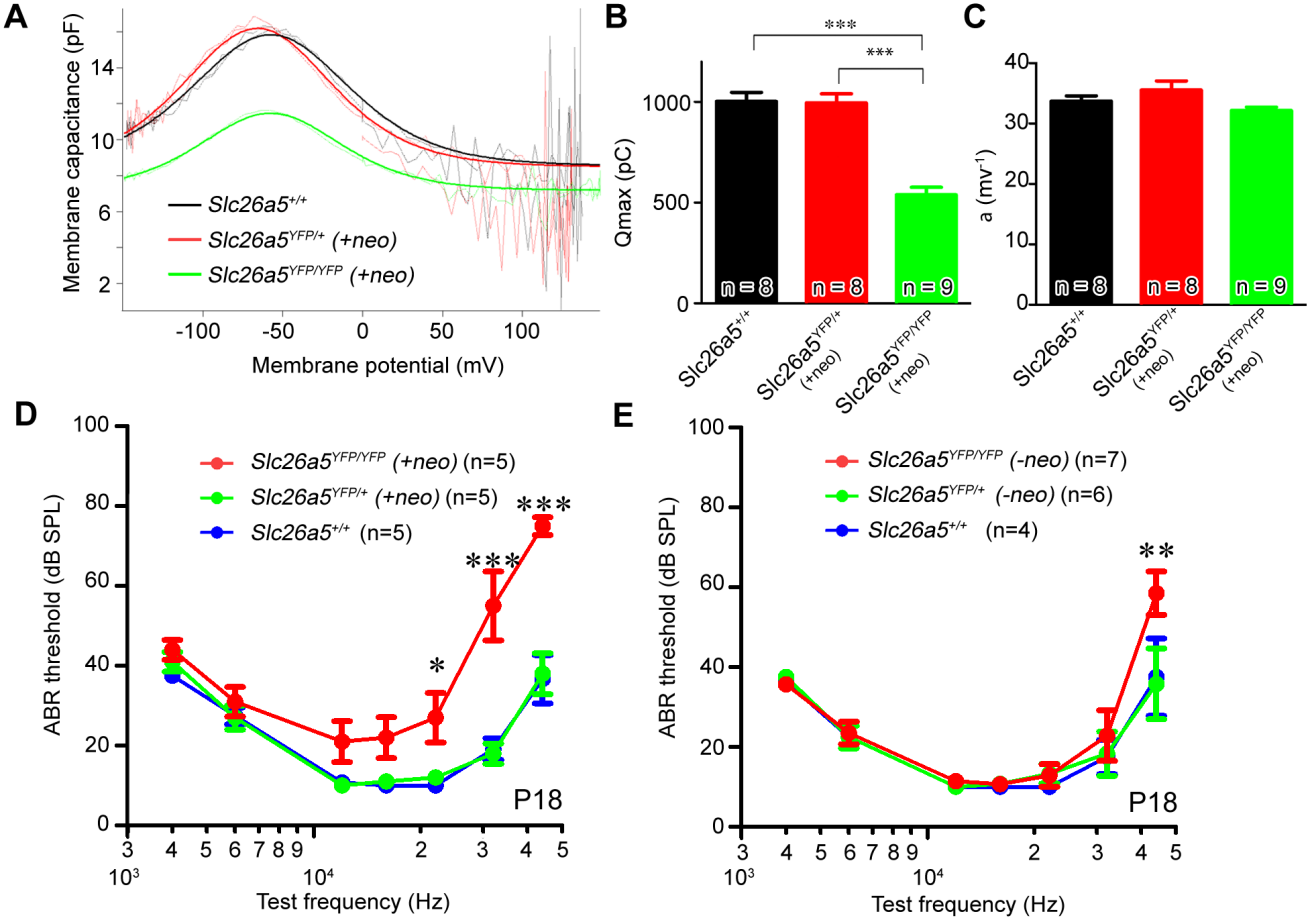
Slc26a5-YFP is functional in Slc26a5-YFP mice *(+neo)*. (A-C) NLC using isolated OHCs from *Slc26a5* ^*YFP/+*^ *(+neo)* mice at P20-P26. (A) NLC in isolated OHCs from *Slc26a5* ^*+/+*^ (black dotted lines), *Slc26a5* ^*YFP/+*^ *(+neo)* (red dotted lines), and *Slc26a5* ^*YFP/YFP*^ *(+neo)* (green dotted lines) cochleae is shown. Smooth lines were obtained by fitting to a second order Boltzmann function where population Q_max_ and α are shown in (B) and (C) respectively. Black (*Slc26a5* ^*+/+*^), red (*Slc26a5* ^*YFP/+*^ *(+neo)*), and green (*Slc26a5* ^*YFP/YFP*^ *(+neo)*) bars express the mean (± S.E.M.). Values are the mean ± S.E.M; (D) ABR thresholds of wildtype and Slc26a5-YFP *(+neo)* mice at P18 are similar (E) ABR thresholds of wildtype and Slc26a5-YFP *(-neo)* mice at P18 are similar. Values are the mean ± S.E.M.; ***: P<0.001, *: P<0.05 by two-way ANOVA followed by student's t test with a Bonferroni correction.

We determined Slc26a5 function by patch-clamping using isolated OHCs from *Slc26a5* ^*YFP/+*^ *(+neo)* or *Slc26a5* ^*YFP/YFP*^ *(+neo)* mice. These cells exhibited the expected bell-shaped non-linear capacitance in response to changes in membrane potential (Fig 2A). The curves were fitted to a second order Boltzmann function to obtain the total elementary charge movement (Q_max_) and the voltage dependence (α; Fig 2B and 2C). No differences were observed between *Slc26a5* ^*YFP/+*^ *(+neo)* OHCs and *Slc26a5* ^*+/+*^ controls at P20-P26 (ANOVA, P>0.05). *Slc26a5* ^*YFP/YFP*^ *(+neo)* OHCs exhibited functional Slc26a5 activity with wild-type like α (ANOVA, P>0.05, Fig 2C) whereas Q_max_ in *Slc26a5* ^*YFP/YFP*^ *(+neo)* OHCs was reduced to 53.65 ± 0.08% of that in wild-type controls (ANOVA, P<0.05, Fig 2B). These data indicate that the YFP fusion construct does not disrupt Slc26a5 function although the functional Slc26a5 amount was diminished in *Slc26a5* ^*YFP/YFP*^ *(+neo)* OHCs.

To confirm that OHCs functioned normally *in vivo*, we used auditory brainstem response (ABR) measurements to determine the hearing sensitivity. At P18, there was no difference between *Slc26a5* ^*YFP/+*^ *(+neo)* mice and controls (ANOVA, P > 0.05; Fig 2D) while *Slc26a5* ^*YFP/YFP*^ *(+neo)* mice also exhibited normal hearing sensitivity for all frequencies except at and above 22kHz (ANOVA, P > 0.05; Fig 2D). It should be noted that *Slc26a5* ^*-/-*^ mice exhibit loss of hearing sensitivities compared to wild-type up to 40 to 60 dB between 4–44 kHz [3, 7, 8, 18]. The high-frequency hearing loss observed in *Slc26a5* ^*YFP/YFP*^ *(+neo)* is most likely due to the presence of a *neo cassette* at the *Slc26a5-YFP* locus. To confirm this, we removed the *neo cassette* in Slc26a5-YFP mice [*Slc26a5* ^*YFP*^ *(-neo)*] (see S1 Text) and measured the hearing sensitivity (Fig 2E). No differences were observed at P18 in *Slc26a5* ^*YFP/+*^ *(-neo)* mice compared to *Slc26a5* ^*+/+*^ mice at any of the tested frequencies; *Slc26a5* ^*YFP/YFP*^ *(-neo)* mice also exhibited normal hearing sensitivity for all frequencies tested up to 32 kHz (ANOVA, P>0.05; Fig 2E). These results (Fig 2) demonstrate that Slc26a5-YFP is functional and does not have a dominant function *in vivo* although Slc26a5-YFP could not be perfectly replaced with endogenous Slc26a5. Therefore, we focused hereafter our analyses on *Slc26a5* ^*YFP/+*^ *(+neo)* mice.

### Limited Slc26a5-YFP lateral diffusion in the lateral wall of OHCs

**Fig 3:**
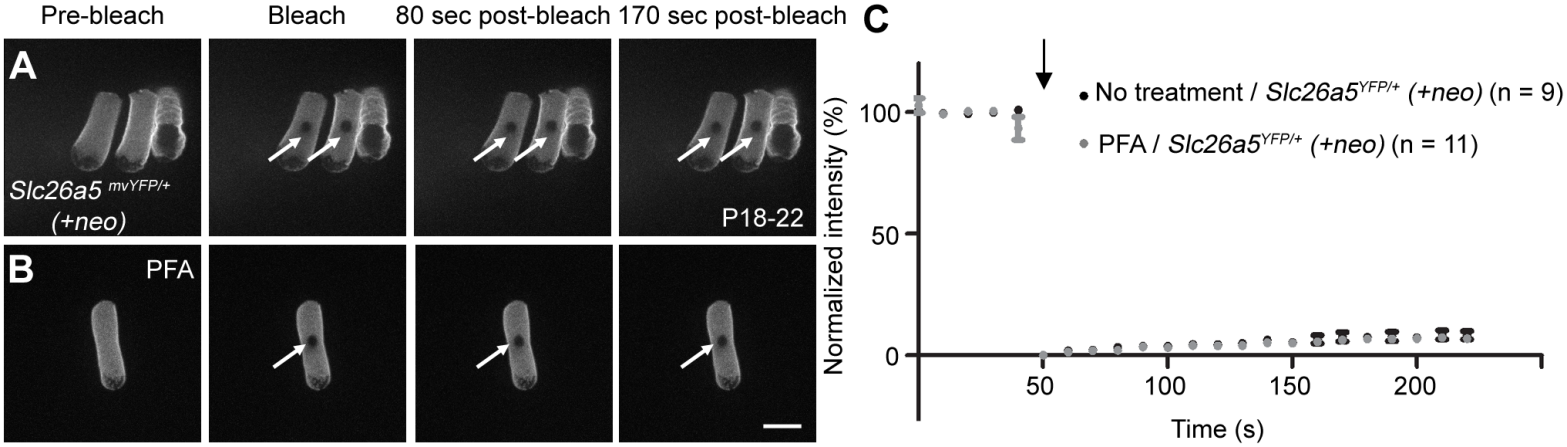
FRAP analysis reveals that Slc26a5 has little lateral mobility in the isolated *Slc26a5* ^*YFP/+*^ *(+neo)* OHCs. FRAP examples using the mice at P18-22 for untreated (A) and PFA-treated (B) OHCs are shown. (C) The normalized fluorescence recovery curves for Slc26a5-YFP based on analysis of the bleached spots (see Materials and Methods). White arrows (A-B) show bleached spots and the black arrow (C) indicates the time of bleaching. Error bars express S.E.M. Scale bar expresses 10 μm. Numbers (n) of OHCs used in two mice from two litters were shown.

Having established that Slc26a5-YFP mice had a normal distribution of Slc26a5 both as adults and during development and that their OHC function and hearing sensitivity were normal, we proceeded by measuring Slc26a5 mobility using fluorescence recovery after photobleaching (FRAP; Fig 3), a technique [10, 19] where small areas in the lateral wall of *Slc26a5* ^*YFP/+*^ *(+neo)* OHCs are photobleached while measuring the fluorescence intensity (see Materials and Methods). Fig 3A represents YFP fluorescence recovery in isolated OHCs from *Slc26a5* ^*YFP/+*^ *(+neo)* mice at P18-22. No significant recovery was observed over 170 s after photobleaching (ANOVA, p> 0.05, Fig 3; n = 9). Additionally, no significant recovery of fluorescence was observed in *Slc26a5* ^*YFP/YFP*^ *(+neo)* OHCs over 170 sec after photobleaching (ANOVA, p>0.05, S4 Fig), demonstrating that functional Slc26a5-YFP is immobile in both hetero- and homozygous animals. Because photobleaching of GFP-like fluorescent proteins does not necessarily destroy fluorescence [20], we also incubated the isolated OHCs from Slc26a5-YFP mice in 4% paraformaldehyde for 20 min, to create an immobile control cell (n = 11). Again, no recovery of fluorescence was observed.

To independently validate this finding, we used brightness and number analysis [21–24]. This method uses fluorescence fluctuations over time to determine whether molecules are mobile. In brief, if we repeatedly count the number of photons emitted during a fixed time interval from Slc26a5-YFP, the count rates will fluctuate randomly around the mean value according to the Poisson distribution, which is characterized by a variance (σ^2^) of the count rate that equals its mean value (μ)–as long as molecules remain fixed within the focal volume of the lens (σ^2^/μ = 1). If molecules are moving, an additional source of fluorescence variation is introduced as molecules diffuse in and out of the detection volume (σ^2^/μ > 1). Hence, the σ^2^/μ ratio can be used to separate mobile from fixed fluorophores [14].

**Fig 4:**
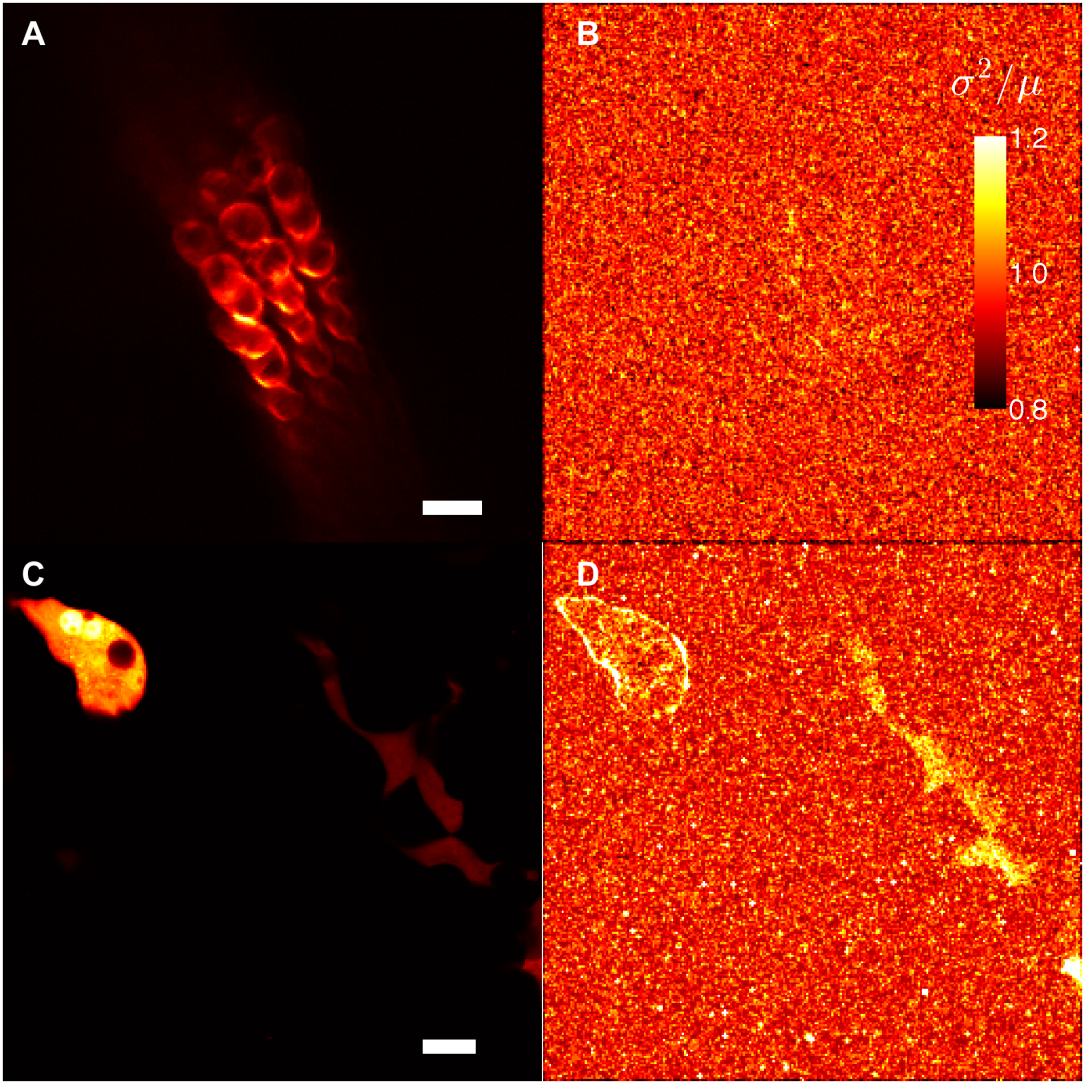
B & N analysis confirms the minimal lateral mobility of Slc26a5-YFP in *Slc26a5* ^*YFP/+*^ *(+neo)* mice. (A) YFP fluorescence distribution in a temporal bone preparation from a *Slc26a5* ^*YFP/+*^ *(+neo)* mouse at P38 is shown. (B) Variance of the image series (225 frames) divided with the average brightness (σ^2^/μ) is shown corresponding to A. (C) YFP fluorescence distribution in Min6m9 cells expressing cytosolic YFP (see Materials and Methods) is shown. The image was taken under an identical condition with (A). B & N analysis for cytosolic YFP in Min6m9 cells (C) is shown in (D). Scale bars express 20 μm.

A structural image of YFP fluorescence in a temporal bone preparation from *Slc26a5* ^*YFP/+*^ *(+neo)* mice is shown in Fig 4A. A map of σ^2^/μ shows that OHCs cannot be distinguished from the background (Fig 4B). The mean value of σ^2^/μ in the OHCs of 5 separate acquisitions from 4 different preparations was 1.009 ± 0.003, whereas the corresponding values for the surrounding pixels was 1.002 ± 0.0003 (paired t-test, P>0.05; for a photon-counting detector as the one employed here, σ^2^/μ will equal 1 in background pixels; [22]). For comparison, fluctuations of heterologously expressed cytosolic YFP in Min6m9 cells were measured (Fig 4C). The diffusion of the fluorescent molecule is expected to result in higher variance; a map of σ^2^/μ indeed shows that cells can be distinguished from the background (Fig 4D). These results together confirmed that diffusion of Slc26a5-YFP is minimal in the lateral wall of OHCs.

### Co-treatments of Methyl-β-cyclodextrin (MβCD) and salicylate collapse membrane-associated structures and increase Slc26a5-YFP mobility

**Fig 5:**
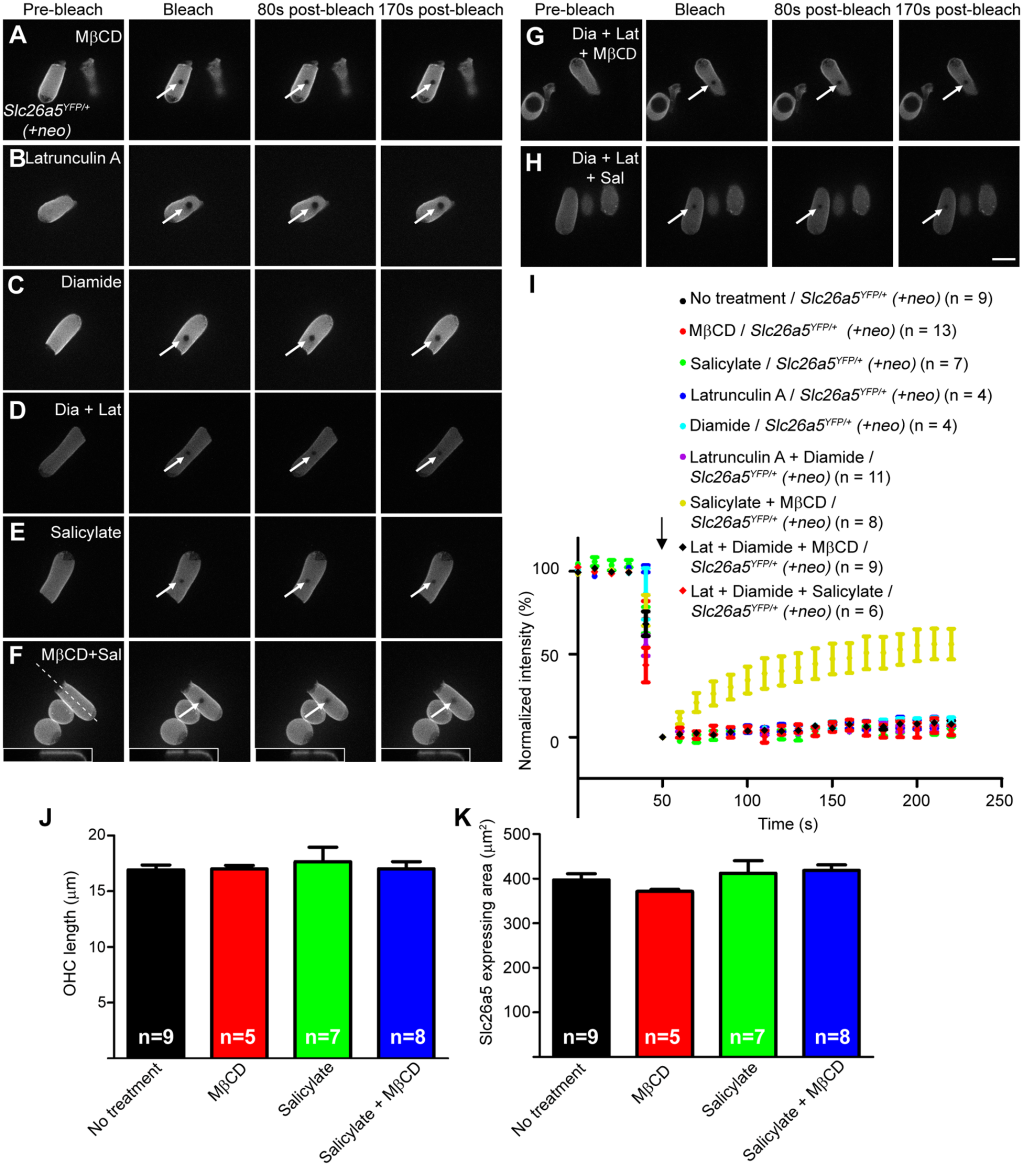
Slc26a5 mobility analysis in the lateral wall of isolated OHCs at P18-22. FRAP examples for MβCD-treated (A), latruculin A-treated (B), diamide-treated (C), latrunculin A/ diamide-treated (D), salicylate-treated (E), MβCD/salicylate-treated (F), diamide/latrunculin A/MβCD-treated (G), diamide/latrunculin A/salicylate-treated (H) OHCs from *Slc26a5* ^*YFP/+*^ *(+neo)* mice are shown. Scale bar expresses 10 μm. The dashed line in (F) indicates the position of optical section shown in insets. (I) The normalized fluorescence recovery curves for images A-H are shown (see Materials and Methods). White arrows in A-H show bleached spots and the black arrow in I indicates the time of bleaching. Error bars express S.E.M. OHC length (J) and Slc26a5 expressing area (K) after each treatment is shown. Values are the mean ± SEM.

To ascertain factors that modulate Slc26a5’s lateral diffusion, we used pharmacological treatments to manipulate membrane fluidity and membrane-associated structures in isolated OHCs (Fig 5). Cholesterol is known to restrict membrane fluidity and depletion of membrane cholesterol induced increased confinement areas of Slc26a5-GFP in 293T cells, presumably by disrupting membrane classical raft [11]. Furthermore, depletion of membrane cholesterol altered cochlear mechanical amplification *in situ* [25] and *in vivo* [26]. To address whether depletion of cholesterol makes Slc26a5 mobile, OHCs from *Slc26a5* ^*YFP/+*^ *(+neo)* mice were incubated with MβCD, which reduces cellular cholesterol (20–60 min incubation time); however, no significant FRAP recovery was observed over 170 sec after treatment with MβCD alone (ANOVA, p> 0.05) (Fig 5A and 5I). Using the common classical raft marker GM1 [27], wild-type OHCs exhibited limited and hardly detectable staining both before and after MβCD treatment (S5A Fig), suggesting that membrane classical rafts are absent from OHCs and therefore cannot contribute to controlling Slc26a5 mobility.

Directly underneath the OHC PM is a cytoskeletal network consisting of actin and spectrin (Fig 1A). Treatment with 1 μM Latrunculin A (actin filament inhibitor [28]), 1 mM Diamide (actin-spectrin binding inhibitor [29]) or both had no effect on fluorescence recovery over 170 s (Fig 5B–5D and 5I and S5B–S5F Fig; ANOVA, p> 0.05). Latrunculin A has been reported to disrupt F-actin in OHC lateral wall in cochlear explant culture at P0 and P3 [28] and diamide has been reported to reduce OHC stiffness [29]. In addition, when OHCs were treated with 5 μM Y-27632 (a RHO inhibitor [30]), no change in Slc26a5’s lateral mobility was observed (S5G and S5H Fig). These results suggest that cytoskeletal structures alone are unlikely to control Slc26a5’s lateral mobility.

**Fig 6:**
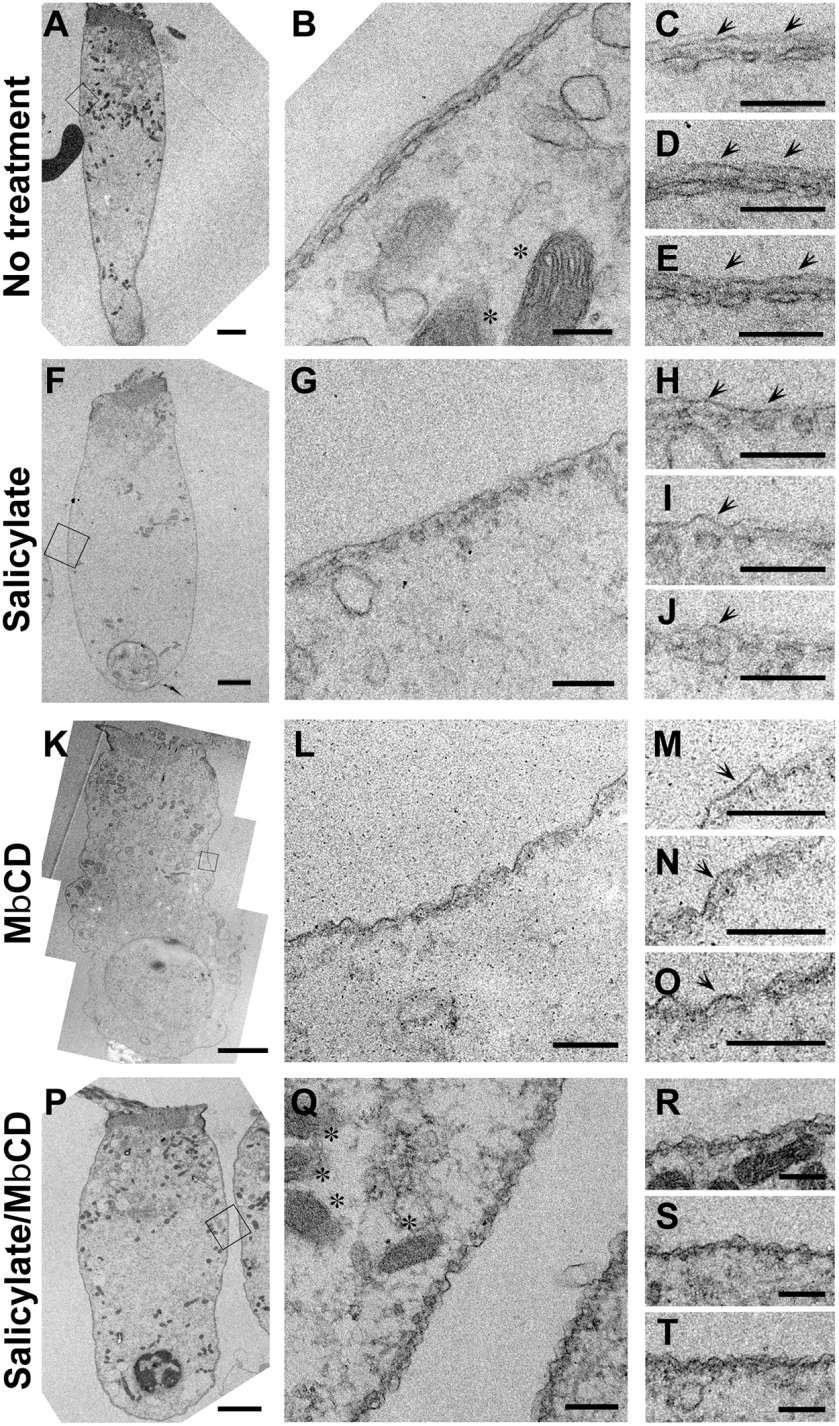
Ultra-structural analysis of the OHC lateral wall in isolated OHCs. TEM studies in untreated (A-E), salicylate-treated (F-J), MβCD-treated (K-O) salicylate/MβCD-treated (P-T) isolated OHCs from the mice at P22-23 are shown. Black squares in A, F, K, P were enlarged in B, G, L, and Q, respectively. Asterisks indicate mitochondria. Arrows indicate assumptive pillars. Scale bar expresses 2 μm in A, F, K, and P and 200 nm in B-E, G-J, L-O and Q-T.

We further examined ultrastructure of OHC lateral walls of isolated OHCs under drug treatments (Fig 6). The untreated OHC (the control) maintained a cylindrical shape and smooth appearance of cell membrane with clear appearance of mitochondrial inner and outer membrane, indicating a healthy cell (Fig 6A–6E). Good OHC integrity and viability were also seen even after exposure with either salicylate alone, MβCD alone, or salicylate in combination with MβCD for 20 min under identical conditions, as indicated by presence of clear appearance of PM and mitochondrial double membrane (Fig 6F, 6K, 6P, 6Q and 6R). Previously, it has been shown that salicylate reversibly vesiculates SSC in a time- and dose-dependent manner [31, 32]. Consistently, the isolated OHC after salicylate treatment exhibited fenestrated SSC retaining smooth appearance of cell membrane while the OHC with no treatment exhibited largely continuous SSC (Fig 6B and 6G). Cell membranes in MβCD alone and salicylate/MβCD-treated OHCs became wavier and more undulated compared to those in untreated and salicylate-treated OHCs (Fig 6K–6T). The space between PM and SSC was less defined in MβCD treated cells and essentially vanished in salicylate/MβCD-treated OHCs (Fig 6K–6T). Notably, assumptive pillar structure was almost exclusively missing in salicylate/MβCD-treated OHCs (n = 4 from two mice) while fully intact in untreated and salicylate-treated OHCs, and compromised in many but a few selected cases for MβCD-treated OHCs (Fig 6C–6E, 6H–6J, 6M–6O and 6R–6T). These data indicated that salicylate treatment in combination with MβCD to isolated OHCs collapsed trilaminate organization (e.g. disruption of the extracisternal space (ECiS)) in OHC lateral wall but SSC and PM still remained although these studies cannot fully exclude the possibility of other changes such as alterations in biophysical or biochemical properties of the PM.

**Fig 7:**
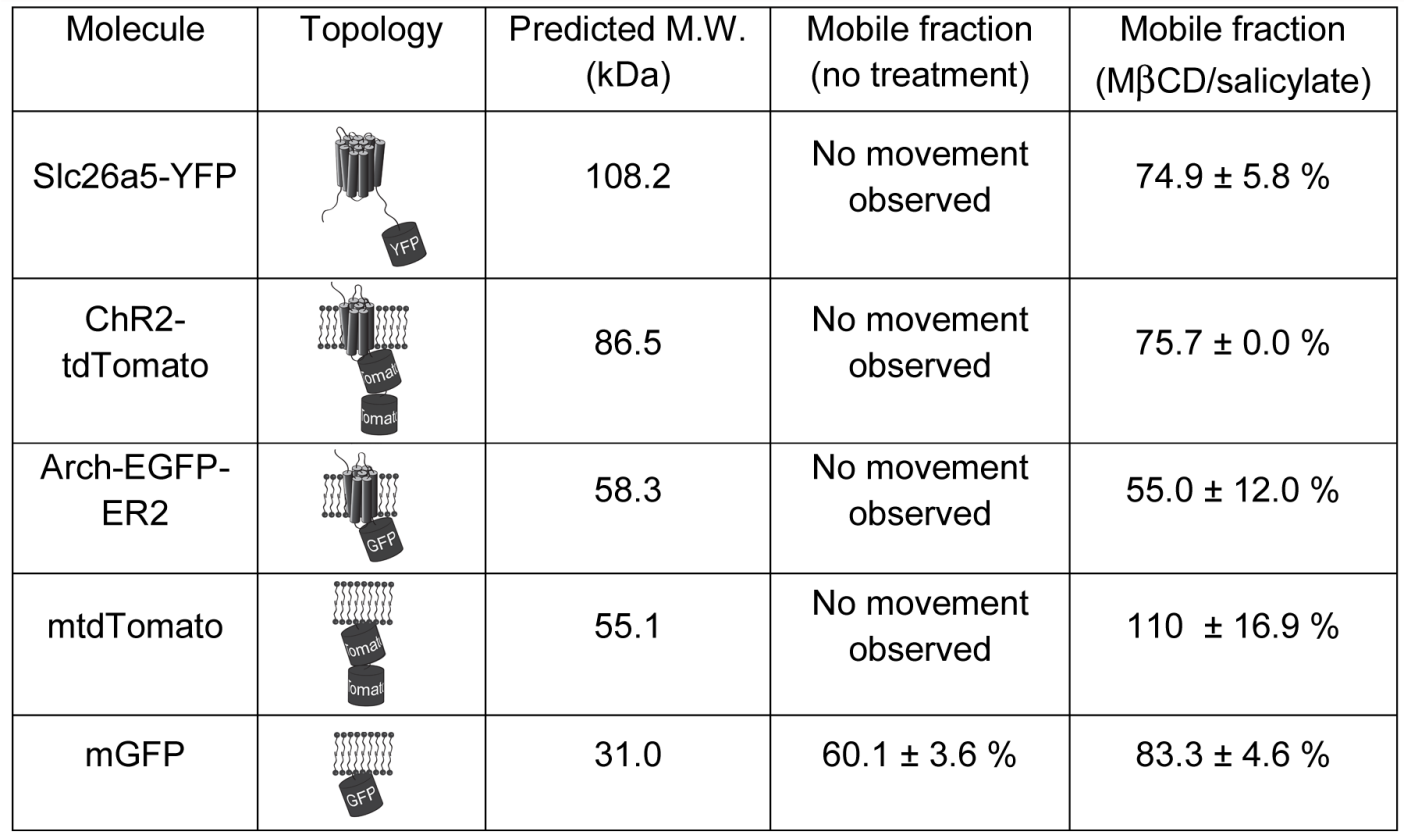
Topology, molecular weight, and mobile fractions before and after treatment of Slc26a5 and four heterologously expressed membrane proteins. Mobile fraction for each molecule was shown either before or after co-treatment of MβCD and salicylate. Molecular weight (M. W. in kDa) was predicted based on amino acid sequences. Means and S.E.M. are expressed.

Because salicylate treatment in combination with MβCD appeared to collapse arranged trilaminate structure in OHC lateral wall, we performed FRAP analysis on cells treated with salicylate alone and salicylate in combination with MβCD. Salicylate alone did not change Slc26a5’s mobility (Fig 5E and 5I) as no significant recovery of fluorescence was observed over 170 sec (ANOVA, P>0.05), but incubation of *Slc26a5* ^*YFP/+*^ *(+neo)* OHCs with both MβCD and salicylate led to significant recovery after photobleaching (74.9 ± 5.8% recovery; ANOVA, P<0.05, Figs 5F and 5I and 7). The lateral diffusion was observed only at the PM (Fig 5F, insets). Previously, we demonstrated that reduced density of Slc26a5 caused shorter OHC in length [33]. Therefore, lengths of OHCs used for FRAP experiments were measured. Notably, no differences were observed in OHC length after treatment with either MβCD alone, salicylate alone, or salicylate in combination with MβCD (Fig 5J; ANOVA, p> 0.05). In support, Slc26a5’s expressing area of OHCs used for FRAP experiments were also similar (Fig 5K; ANOVA, p> 0.05). Although due to technical difficulties, we could not measure OHC capacitance electro-physiologically using isolated OHCs after treatment of salicylate in combination with MβCD, these results support the idea that incubation time of these drug-treatments under conditions used were too short for a significant change in Slc26a5 density. Therefore, these results argue against the idea that reduced Slc26a5 density liberated constrained lateral diffusion of Slc26a5. In contrast, combinations of either MβCD or salicylate with Latrunculin A, Diamide, or both failed to make Slc26a5-YFP mobile (Fig 5G–5J; ANOVA, p> 0.05). These results suggest that the integrity of arranged trilaminate structure (e.g. presence of the ECiS) is the major impediment for Slc26a5 mobility in the OHC lateral wall.

To further define the relation between Slc26a5 and the trilaminate structure, we analyzed the ultrastructure of the OHC lateral wall in *Slc26a5* ^*-/-*^ mice. The SSC appeared intact in *Slc26a5* ^*-/-*^ mice, similar to that in *Slc26a5* ^*+/+*^ mice (S6A and S6B Fig), consistent with a previous report [34]. Thus, while Slc26a5 is not required for the integrity of trilaminate structure, the integrity of trilaminate structure is critical for Slc26a5’s mobility within the OHC lateral wall.

### Channelrhodopsins and other membrane-bound fluorescent proteins exhibit minimal mobility in OHCs

To test the mobility of membrane proteins other than Slc26a5, we heterologously expressed in mice (see S1 Text) each of four Slc26a5-unrelated membrane proteins (Channelrhodopsin-2(H134R)–tdTomato (ChR2-tdTomato), Arch-EGFP-ER2, membrane-GFP (mGFP), and membrane-tdTomato (mtdTomato) [35, 36]. The channelrhodopsins are seven-transmembrane-helix proteins with fluorescent proteins fused at their C-termini and predicted molecular weights of 86.5 kDa for ChR2-tdTomato and 58.3kDa for Arch-EGFP-ER2 (Fig 7). In comparison, Slc26a5 has 14 transmembrane domains and a putative molecular weight of approximately 75.0 kDa [37] while Slc26a5-YFP is predicted to be 108.2 kDa (Fig 7). In addition, mGFP and mtdTomato bind to the inner leaflet of lipid bilayer with predicted molecular weight of 31.0 and 55.1 kDa, respectively (Fig 7).

**Fig 8:**
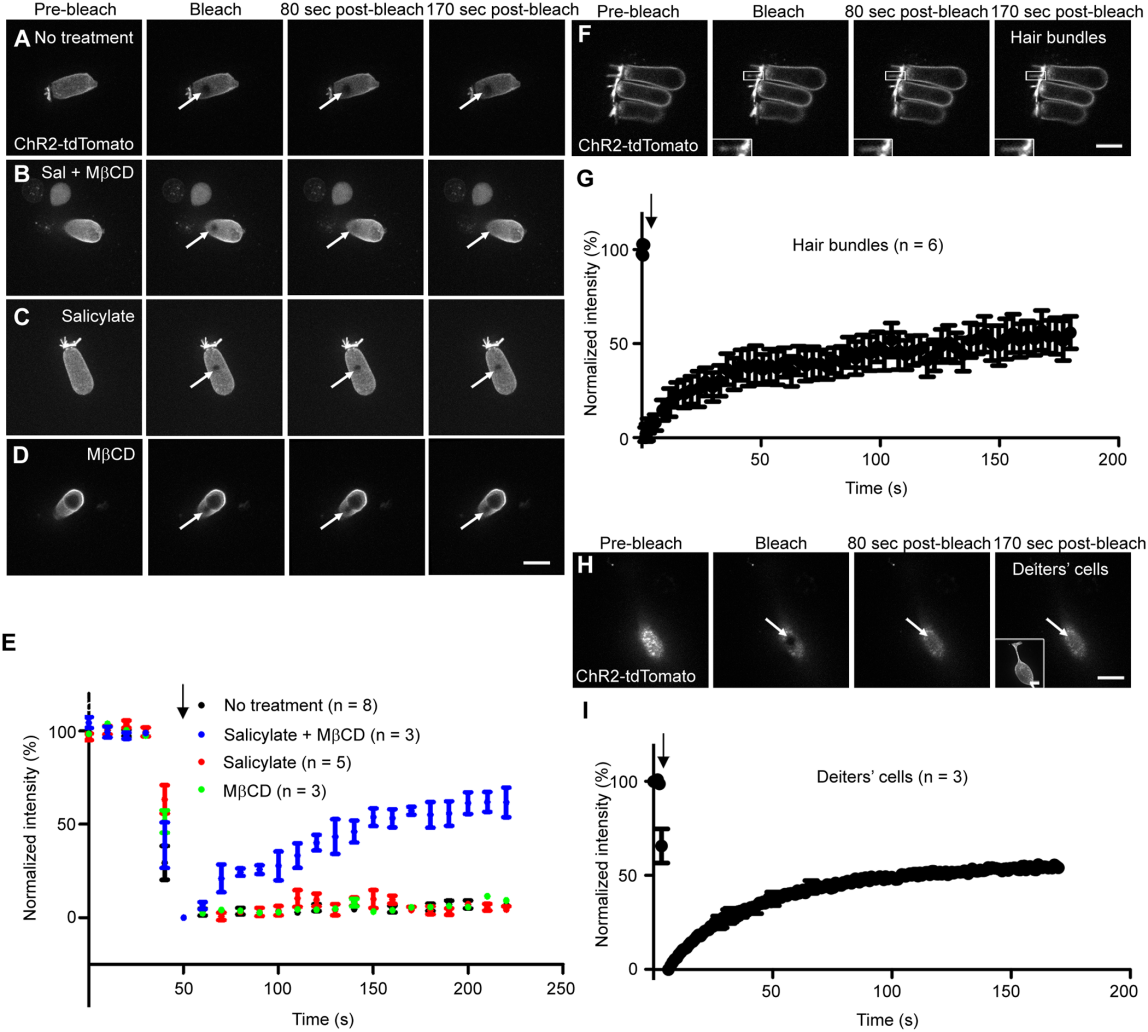
Mobility of ChR2-tdTomato in isolated OHCs and SCs at P18-22 using FRAP analysis. (A-D) ChR2-tdTomato heterologously expressed OHCs are shown. Untreated (A), salicylate/MβCD-treated (B), salicylate-treated (C), and MβCD-treated (D) OHCs from *Slc26a5-CreER* ^*T2+/-*^;*ChR2-tdTomato* ^*f/+*^ mice after tamoxifen was intraperitoneally injected at P6-7 are shown. (E) The normalized fluorescence recovery curves for A-D are shown. (F) ChR2-tdTomato mobility in OHC hair bundles is shown and white-squared areas with hair bundles are shown in the insets. Initial ten data points for ChR2-tdTomato were taken at 0.248 second intervals and the rest of 60 data points were taken at 3 second intervals. (G) The normalized fluorescence recovery curve as for image F is shown. (H) ChR2-tdTomato mobility in Deiters’ cell PM is shown. The inset shows the cell after FRAP obtained by maximum intensity projection of z-stack. Deiters’ cells were isolated from *FgfR3-iCreER* ^*T2+/-*^;*ChR2-tdTomato* ^*f/+*^ mice intraperitoneally injected with tamoxifen at P6 and P7 (see Materials and Methods). Each data point was taken at 0.843 second intervals. (I) The normalized fluorescence recovery curve as for image H is shown. Values are the mean ± S.E.M. Scale bars express 10 μm. White arrows (A-D, F, and H) show bleached spots and black arrows indicate the time of bleaching. Numbers (n) of cells analyzed in more than three mice are shown.

When ChR2-tdTomato was specifically expressed in OHCs (see S1 Text), the compound knockin mice exhibited normal hearing at all frequencies tested (4–44 kHz, S7A Fig), demonstrating that ChR2-tdTomato does not interfere with either Slc26a5 or OHC function. tdTomato expression was observed in the entire OHC membrane, including the hair bundles (Fig 8A). In the lateral wall of OHCs, ChR2-tdTomato was immobile up to 170 sec (Figs 7 and 8A and 8E) but treatment with MβCD and salicylate liberated the protein, leading to a mobile fraction of 75.7% (ANOVA, P<0.05, Figs 7 and 8B and 8E). Neither salicylate nor MβCD alone had any effect on ChR2-tdTomato mobility (ANOVA, P>0.05, Fig 8C–8E). These results are quite similar to those with Slc26a5-YFP. It is possible that minimal lateral diffusion of Slc26a5 reduces mobility of other membrane proteins. To test this, we heterologously expressed ChR2-tdTomato in OHCs of *Slc26a5* ^*-/-*^ mice, but failed to isolate healthy OHCs from these mice, likely due to the fragile nature of *Slc26a5* ^*-/-*^ OHCs.

To examine ChR2-tdTomato’s mobility in other membranes, we examined ChR2-tdTomato diffusion in OHC hair bundles and Deiters’ cells (adjacent to OHCs) of different knockin mice (see Materials and Methods). 59.6 ± 8.5% of ChR2-tdTomato was mobile in OHC hair bundles (Fig 8F and 8G). Similarly, 64.8 ± 1.2% of ChR2-tdTomato was mobile (Fig 8H and 8I) in the Deiters cell membrane.

Together, all four heterologously expressed membrane fluorescent proteins were relatively immobile in the OHC lateral wall and the combined treatment of MβCD and salicylate increased their mobility in the lateral wall of OHCs (Figs 7 and 8 and S7 and S8 Figs).

## Discussion

### Structural roles of OHC lateral wall

We have shown that the mobility of membrane proteins is constrained by the trilaminate structure of the OHC lateral wall. We hypothesize that Slc26a5 forms a motor complex with membrane-associated structures including PM, the SSC, and assumptive pillar structures (S9 Fig). This motor complex tethers Slc26a5, providing a route for Slc26a5-generated force to be transmitted throughout the cell, subsequently contributing to increasing the sensitivity and frequency resolution of the hearing organ.

Several lines of evidence suggest structural roles of Slc26a5 in OHCs *in vivo*. First, OHCs from *Slc26a5* ^*-/-*^ mice are shorter in length than those of wildtype controls [7], but this reduction was abolished in mice where non-functional Slc26a5 was present in normal amounts [4]. Previously, we also demonstrated that OHC length was reduced in a Slc26a5-dose-dependent manner in the region corresponding to 4–22 kHz [33]. Second, the somatic stiffness in *Slc26a5* ^*-/-*^ OHCs was decreased as compared to wildtype controls [4]. The decreases were eliminated in the mice lacking functional Slc26a5 but possessing identical amount of Slc26a5 [4]. *In vivo* basilar membrane displacement measurements of Slc26a5-null and non-functional Slc26a5 mice further support Slc26a5’s structural role [38]. These studies suggest that Slc26a5 is a structural protein that adjusts the mechanical properties of OHCs to produce the correct cochlear impedance. In this study, we showed that pharmacological disruption of membrane-associated structures liberates Slc26a5, which began to diffuse. Moreover, additional membrane associated fluorescent proteins were also immobile, likely further strengthening Slc26a5’s structural role. Additionally, we showed that Slc26a5 was not required for membrane integrity of OHC lateral wall. These findings thus provide new insights on how trilaminate structure contributes to structural roles of OHC lateral wall through Slc26a5.

In support of minimal lateral diffusion of Slc26a5 we report here, we note that the protein turnover rate in both stereocilia and the lateral wall of OHCs is extremely low, as no significant exchange of actin monomers was observed for 7 days *in vivo* [39]. In addition, debris of OHCs containing Slc26a5 were detected within supporting cells at least 9 days after phagocytosis [40], demonstrating that Slc26a5 has a long half-life.

Slc26a5-YFP immobility may appear contradictory to previous predictions based on: 1) Slc26a5-GFP mobility measurements in heterologously over-expressed 293T cells [12]; and 2) NLC measurements in isolated OHCs with inactivated Slc26a5 in a certain small spot [13] where the estimated Slc26a5 diffusion coefficiency was 0.03 to 0.063 and 0.08 to 0.35 (μm^2^/s), respectively. These results, however, may be entirely consistent with ours reported here for several reasons. (1) There is no trilaminate structure in the PM of 293T cells and the lipid content of 293T cells is different from that in the OHC lateral wall (i.e., the likely absence of classical rafts), thus supporting the importance of the laminated structure of OHC lateral wall. (2) Because Lucifer yellow injected into OHCs can diffuse everywhere inside of OHCs and the OHCs were exposed by light over 10 min within a small spot, it is possible that free radicals created by laser inactivation simultaneously disrupt other OHC structures and functions closely associated with Slc26a5 (i.e., trilaminate structure) [13]. (3) Repetitive voltage stimulation to indirectly measure Slc26a5’s mobility by photoexposure of Lucifer yellow using fiber optic light could, in combination, further damage OHCs [13]. (4) Similarly, our experiments have limitations in that they were carried out at steady state (i.e. not under membrane alternating voltage stimulation that produces electromotility). Therefore, we could not address the possibility that Slc26a5 might diffuse when OHCs contract and elongate in length in response to membrane voltage changes in our current setup as seen in a previous report [13].

### The integrity of the trilaminate structure is the main determinant of minimal lateral diffusion of membrane proteins in the OHC lateral wall

Diffusion of proteins in cell membranes is generally lower than in artificial membranes due to molecular crowding (high protein density), intrinsic membrane properties (fluidity and microdomains), and/or cytoskeleton network underneath the PM [41]. Given that depletion of membrane cholesterol in the presence of MβCD and salicylate turned Slc26a5 more mobile after 20 min of treatment, which is too short for a significant change in Slc26a5 density, molecular crowding is unlikely to be a major determinant of Slc26a5 mobility. Our results thus do not support the notion that Slc26a5 is so tightly packed that the lateral wall of OHCs has no extra space for lipids or other membrane proteins [1]. In support, four different membrane fluorescent proteins can be heterologously expressed in the lateral wall of OHCs and yet do not interfere with Slc26a5’s normal function.

Co-treatments of MβCD and salicylate make not only Slc26a5 but also four unrelated, heterologously expressed membrane fluorescent proteins (ChR2-tdTomato, Arch-EGFP-ER2, mGFP, and mtdTomato) more mobile in the OHC lateral wall, despite differences in amino acid sequences, molecular sizes and numbers of transmembrane domains. Moreover, heterologously expressed ChR2-tdTomato was immobile in the OHC lateral wall where the trilaminate structure is present but clearly mobile in OHC stereocilla and nearby supporting cell membranes. These surprising results highlighted several molecular and structural factors that are critical for Slc26a5’s lateral mobility in OHC lateral wall and, ultimately, function. First, depletion of membrane cholesterol by MβCD increases membrane fluidity and thus lateral mobility of membrane proteins in the lateral wall of OHCs, despite that cholesterol level is relatively low in the lateral wall of OHCs [26]; however, unlike Slc26a5-transfected 293T cells, PM classical rafts are insignificant in the OHC lateral wall [42]. In support, Slc26a5 was not found in GM1- and cholesterol- enriched fraction in chicken cochlear duct [27], although heterologously expressed Slc26a5-EGFP was detected in membrane raft-enriched fraction in cultured HEK 293 cells [43]. Second, the trilaminate structure of OHC lateral wall most likely plays a role in maintaining the cell’s cytoplasmic turgor pressure and cylindrical shape, both of which are necessary for the cell’s active and passive contribution to cochlear mechanics [44]. Thus, collapsed trilaminate structure by both salicylate and MβCD can remove a key constraint on membrane proteins in the lateral wall of OHCs. In support, OHC electromotility is diminished and ultimately abolished when OHCs become flaccid [2, 45, 46], and polystyrene microspheres labeled membrane in lateral wall become re-oriented towards cell’s longitudinal axis when a quiescent OHC is electrically stimulated [30]. Together, these results strongly support that the integrity of the organized trilaminate structure constitutes the main factor that coordinate the orientation of the Slc26a5 motor units in the OHC lateral wall and endow the greater axial than radial electromotile response.

### Implications on cellular motors and human diseases

We note that structures found in OHCs—a sandwich of immobile membrane proteins, cytoskeleton and endoplasmic reticulum forming a trilaminate organization—is found in other cells exposed to high mechanical stress levels or with a requirement for fast signaling. Examples include skeletal muscle, the initial segments of axons, gliding bacteria, and plant cell walls [47–52]. Possibly, this may represent an example of convergent evolution of cellular features across the spectrum of life.

Interestingly, Niemann-Pick Disease Type C (NPC) is characterized by intralysosomal accumulation of cholesterol [53, 54]. Administration of 2-hydroxypropyl-β-cyclodextrin (HPβCD, an analog of MβCD) resulted in measurable graded and rapid onset of high frequency hearing loss in both NPC patients and animal models [55, 56]. Our findings here that co-treatment of MβCD and salicylate collapses trilaminate organization within 20 minutes and liberates otherwise anchored Slc26a5 suggest a mechanism of trilaminate organization defects in NPC that warrants future studies.

## Materials and Methods

### Ethics statement

The Animal Care and Use Committees of St. Jude Children’s Research Hospital (approval number 319), and Regional Ethics board in North Stockholm approved all of the protocols performed in this study. Mice were housed under a 12 h light/dark cycle with free access to food and water. Toe clipping and genotyping was performed in accordance with *Guide for the Care and Use of Laboratory Animals*.

### Fluorescent recovery after photobleaching (FRAP) using isolated OHCs

All FRAP experiments using isolated OHCs in this study was prepared from mice at postnatal 18–22 days of age. Dissociation of OHCs was described in Supplementary Methods. The isolated OHCs were put in the Petri dishes. The extracellular solution used was 155mM NaCl, 4mM KCl, 2mM CaCl_2_, 1mM MgCl_2_, 10mM HEPES. The osmolarity and pH was adjusted to 320–330 mOsm/kg and 7.3. A criterion of healthy OHCs was cylinder-shaped. Although internalization of membrane inside of OHC was occasionally observed, those cells were not chosen. Occasionally, OHCs became swollen in the course of experiments. These data were discarded. All of data were recorded within two hours after sacrificing mice. FRAP experiment was performed using Spinning Disk Confocal microscope system (Intelligent Imaging Innovations, Inc., Denver, CO, USA) with a Zeiss Axio Observer Z1 (Zeiss) equipped with a Plan-Apochromat 63 × oil immersion and 1.4 NA objective (Zeiss), Yokogawa CSU-X1 Spinning Disk (Yokogawa, Tokyo, Japan), 3iLaser stack (Intelligent Imaging Innovations, Inc.) and Evolve 512 EMCCD camera (Photometrics Ltd., Tucson, AZ, USA). Photobleaching was performed with a circular spot using 514 nm laser for Slc26a5-YFP, 488 nm laser for Arch-EGFP-ER2 and mGFP, 561 nm laser for ChR2-tdTomato and mtdTomato. The laser power for photobleaching was adjusted to photobleach approximately 50–75% fluorescence intensity. Each image was captured with 75 ms exposure time for Slc26a5-YFP in *Slc26a5* ^*YFP/+*^ *(+neo)* OHCs, 50 ms exposure time for Slc26a5-YFP in *Slc26a5* ^*YFP/YFP*^ *(+neo)* OHCs, 50 ms for ChR2-tdTomato, 100 ms for Arch-EGFP-ER2, 100 ms for mGFP, and 200 ms for mtdTomato. Each data point was taken at 10 sec intervals. The pixel sizes for X and Y-axis were 0.203 μm.

To obtain fluorescence recovery curves, maximum intensity projection of z-stacks were created and average intensity of photobleached and un-photobleached area was measured using slidebook 5 (Intelligent Imaging Innovations, Inc.). These values were background-subtracted. The intensity of un-photobleached area at each time point was used to correct acquisition photobleach. Normalized intensity at each time (t) was obtained by using the following equation:

$$f(t)=\frac{I-I_{\text{min}}}{I_{\text{max}}-I_{\text{min}}}\times 100$$ (1)

where *I* is the intensity at each data point, *I*_*min*_ is the intensity after photobleaching, and *I*_*max*_ is the average intensity from first five scans before photobleaching. The photobleach efficiency was calculated by *I*_*min*_/ *I*_*max*_ × 100.

The mobile fraction (A) and characteristic timescale for diffusion (τ_D_) was calculated using the Igor Pro 6.1.2.1 (WaveMetrics, Portland, OR, USA). The obtained normalized fluorescence recovery curves from mGFP, and mtdTomato ectopically overexpressed OHCs were fitted to the following equation that was first proposed by Axelrod [57].

$$f(t)=A\frac{e^{-2\tau_{D}}}{t}\left(I_{0}\left(\frac{2\tau_{D}}{t}\right)+I_{1}\left(\frac{2\tau_{D}}{t}\right)\right)$$ (2)

where *t* is the time, and *I*_*0*_ and *I*_*1*_ are modified Bessel functions.

The fluorescence recovery curve for Slc26a5-YFP and Arch-EGFP-ER2 after treatment with MβCD either with or without salicylate did not fit to Eq 2. Therefore, the following single exponential equation was used;

$$f(t)=A(1+e^{-kt})$$ (3)

where A and k are parameters of the curve, and t is time.

After FRAP experiments, fluorescence images were analyzed with Spinning Disk Confocal microscope system as described above and were captured at 0.27 μm intervals from the upper to lower edges. Optical sections were obtained at depth intervals of 0.6 μm. After a 3D reconstruction of isolated OHCs, the OHC lengths and diameters were measured as OHC lengths and diameters drawing a line along Slc26a5 are expressing region, using the Imaris 8. 1. 0. software (Bitplane, Zurich, Switzerland) as shown in S10 Fig. The surface area of the lateral membrane containing Slc26a5 was calculated by *A*_lat_ = π*DL*, where *D* is the diameter and *L* is the length of the membrane containing Slc26a5 as described before [33].

### Pharmacological treatment

The final concentration of salicylate (SIGMA), MβCD (SIGMA), Latrunculin A (SIGMA), Diamide (SIGMA) used in this study was 10 mM, 1mM, 1μM, and 1mM, respectively. Briefly, isolated OHCs were placed in extracellular solution containing drugs indicated in each figure. 20 min after the incubation, protein’s mobility was measured (see above).

### Mouse temporal bone preparation and brightness and number (B & N) analysis

B & N experiments were performed on isolated preparations of the mouse temporal bone. Following induction of anesthesia by intraperitoneal injection of sodium pentobarbital (Apoteket, Stockholm, Sweden), the animal was decapitated and the temporal bone excised and placed in tissue culture medium (140 mM D-gluconic acid, 6.6 mM NaCl, 100 mM CaCl_2_, 3 mM KCl, 5 mM NaH_2_PO_4_, 100 mM MgCl_2_, 5 mM D-glucose, and 5 mM HEPES (298 mOsm, pH 7.3)). The auditory bulla was then opened and the tympanic membrane removed after carefully disarticulating the incudo-stapedial joint. The preparation was mounted in a custom holder. To maintain cellular viability, a thin piece of plastic tubing was inserted into scala tympani after peeling away the round window membrane. The outlet of the tissue culture medium was through a second opening at the apex of the cochlea. This opening also made it possible to visualize the organ of Corti.

Confocal images were with a Leica TCS SP5 II confocal microscope equipped with a 40x 0.8 NA water immersion objective (Leica, Wetzlar, Germany), operating in the photon-counting mode using the HyD detectors. YFP was excited with the 514 nm line of the build in Argon ion laser. Time series, consisting of 225 images, each 256 x 256 pixels, were acquired with a line frequency of 100 Hz and without inter-frame delay. Data analysis was performed using Matlab (The Mathworks, Natick, MA, USA). Briefly, obtained intensity at each data point for each pixel was extracted to obtain the average intensity (<μ>) and the variance (σ^2^). By dividing the variance of the pixel values along the time dimension with the average intensity, a map of mobile molecules within the preparation (σ^2^/μ) were obtained.

### Sample preparation for transmission electron microscopy

For ultrastructural analysis in isolated OHCs, carbon coated-sapphire discs (LEICA) with the pattern of a finder grid were used to identify locations of isolated OHCs on the discs. The sapphire discs were further coated with poly-L-lysine (SIGMA). Isolated OHCs were prepared as described above, placed on the discs, and treated with either 10 mM salicylate, 1 mM MβCD, or both 10 mM salicylate and 1 mM MβCD in extracellular solution described above for 20 min at room temperature. As a control, the OHC was incubated in extracellular solution with no drugs for 20 min at room temperature. The OHCs were fixed using freshly-prepared fixative solution containing 2% glutaraldehyde and 2% paraformaldehyde, and post-fixed with OsO_4_. After dehydration, the discs were embedded with epon resin. The discs were lifted up and peeled off the blocks after the embedding blocks were trimmed. Images of the thick sections were collected for inspection by a JEOL 1200-EX (JEOL, Peabody, MA, USA) electron microscope operated at 100 kV at up to 50,000 x magnification.

## Supporting Information

S1 Fig Slc26a5-YFP knockin mouse strategy. (A) NLC in Slc26a5-transfected 293T cells. YFP-Slc26a5 and Slc26a5-YFP were expressed in 293T cells, individually. NLC from Slc26a5-YFP transfected 293T cells were shown in red and green lines. YFP-Slc26a5 transfected 293T cells exhibit no NLC shown in yellow, blue, and purple dotted lines. The black line and dots show membrane capacitance at different voltages from mock transfected cells. (B) The targeted Slc26a5-YFP knockin allele. Solid rectangles represent exons 11 through 20 of *Slc26a5* gene. A cassette with *YFP* and the *neo*-selectable marker flanked by loxP was inserted right before the termination codon of *Slc26a5* gene. (C) Genomic southern blot analysis of Slc26a5-YFP mice. Genomic DNAs from *Slc26a5* ^*+/+*^, *Slc26a5* ^*YFP/+*^ *(+neo)*, and *Slc26a5* ^*YFP/YFP*^ *(+neo)* tails were digested with Spe I and two specific probes indicated in B were used separately. (D) PCR-based genotyping of *Slc26a5* ^*+/+*^, *Slc26a5* ^*YFP/+*^ *(+neo)*, and *Slc26a5* ^*YFP/YFP*^ *(+neo)* mice using 3 primers is indicated in B as arrows. No loss of body weight was observed in either *Slc26a5* ^*YFP/+*^ *(+neo)* or *Slc26a5* ^*YFP/YFP*^ *(+neo)* mice, when compared to wild-type control. Ratio between wild-type, *Slc26a5* ^*YFP/+*^ *(+neo)*, and *Slc26a5* ^*YFP/YFP*^ *(+neo)* mice from heterozygous intercrosses followed approximately the Mendelian ratio.
(TIF)

S2 Fig Slc26a5-YFP recapitulates endogenous Slc26a5 distribution in *Slc26a5* ^*YFP/+*^ *(+neo)* mice at neonatal stages. (A-D) Slc26a5-YFP fluorescence (green) in the apical (A and C) and basal (B and D) turns of a *Slc26a5* ^*YFP/+*^ *(+neo)* cochlea at P5. The dashed lines in B-D indicate the positions of optical sections shown in the insets. Myo6 (blue) was labeled as a HC marker in C and D. Enriched F-actin (red) was observed in hair bundles of OHCs in C and D. Nuclei (purple) in C and D were labeled in insets. Confocal images in A and B as well as C and D were taken with identical condition. Slc26a5-YFP fluorescence in vestibular system (E-F) and sperm (G-H) from *Slc26a5* ^*YFP/+*^ *(+neo)* mice is indicated in green. F and H shows region corresponding to E and G as differential interference contrast (DIC) images. No YFP epifluorescence were observed in vestibular system and sperm. Scale bars express 200 μm (in F), 20 μm (in A–D, and H).
(TIF)

S3 Fig Slc26a5-YFP recapitulates endogenous Slc26a5 distribution and is functional in *Slc26a5* ^*YFP/YFP*^ *(+neo)* mice. (A-J) Slc26a5-YFP distributions in *Slc26a5* ^*YFP/YFP*^ *(+neo)* mice. (A-C) Slc26a5-YFP fluorescence in the apical turn of the cochleae from *Slc26a5* ^*YFP/YFP*^ *(+neo)* mice at P21 are shown in green. White square in A is enlarged in B. The dashed line in B indicates the position of optical section shown in the inset. Myo7a (red) was labeled as a HC marker in C. Counter-staining of nuclei (blue) was performed using DAPI shown in C. The dashed line in C indicates the position of optical section shown in the inset. The YFP fluorescence signals were observed only in lateral wall of OHCs in cochleae. Slc26a5-YFP fluorescence in apical turn (D and F) and basal turn (E and G) of *Slc26a5* ^*YFP/YFP*^ *(+neo)* cochleae at P5 are shown in green. The dashed line in E-G indicates the position of optical section shown in the inset. Myo6 was labeled as a HC marker in F-G shown in blue. Enriched F-actin (red) was observed in hair bundle of OHCs shown in F-G. Nuclei in F-G were labeled in purple in the inset. Confocal images in D and E as well as F and G were taken under identical conditions. Slc26a5-YFP fluorescence in vestibular system (H-I) and sperm (J-K) from *Slc26a5* ^*YFP/YFP*^ *(+neo)* mice is indicated in green. I and K shows region corresponding to H and J as DIC images. No YFP fluorescence was observed in vestibular system and sperm. Scale bars express 200 μm (A and I), 20 μm (B-G, and K).
(TIF)

S4 Fig Slc26a5 exhibits minimal lateral mobility in the lateral wall of isolated OHCs from *Slc26a5* ^*YFP/YFP*^ *(+neo)* mice at P18-22 using FRAP analysis. Untreated (A) and PFA-treated (B) OHCs are shown. (C) The normalized fluorescence recovery curves for Slc26a5-YFP based on fluorescence analysis of the bleached spots (see Materials and Methods). White arrows (A-B) show bleached spots and the black arrow (C) indicates the time of bleaching. Error bars express S.E.M. Scale bar expresses 10 μm. Numbers (n) of OHCs in two mice from two litters were shown.
(TIF)

S5 Fig GM1 and F-actin distributions and Slc26a5 mobility analysis in the lateral wall of isolated OHCs after treatment with Y-27632. (A) GM1 distribution using Cholera Toxin Subunit B labeling experiments in lived isolated OHCs from wildtype mice at one month old of age is shown (left panel). The represented image is an optical sliced image. Image of bright field for the identical OHC is shown (middle panel). The merged image is shown in right panel. GM1 expression was below detectable range in lateral wall of OHCs. Identical results were observed from two independent mice. (B-E) F-actin distributions using Alexa Fluor 546 phalloidin labeling experiments in none-treated (B), Latrunculin A-treated (C), Diamide-treated (D), and Latrunculin A/ Diamide-treated (E) isolated OHCs from wildtype mice at one month old of age is shown (left panel). (F) Semi-quantitative analysis of Alexa Fluor 546 conjugated phalloidin’s fluorescence in OHC lateral wall from none-treated, Latrunculin A-treated, Diamide-treated, and Latrunculin A/ Diamide-treated isolated OHCs is shown. (G) FRAP examples for Y-27632-treated OHCs from *Slc26a5* ^*YFP/+*^ *(+neo)* mice at P18-22 are shown. Scale bar expresses 10 μm. (H) The normalized fluorescence recovery curves for images B are shown (see Materials and Methods). White arrows in G show bleached spots and the black arrow in H indicates the time of bleaching. Error bars express S.E.M. Numbers (n) of cells analyzed in two independent mice are shown.
(TIF)

S6 Fig The SSC is present in OHCs from *Slc26a5* ^*-/-*^ mice. Ultrastructure of OHC lateral wall from *Slc26a5* ^*+/+*^ (A) and *Slc26a5* ^*-/-*^ (B) mice are shown. Arrows indicate CL and SSC. Scale bar = 200 nm.
(TIF)

S7 Fig ABR thresholds of *Slc26a5CreER* ^*T2 +/-*^;*ChR2-tdTomato* ^*f/+*^, *Slc26a5CreER* ^*T2 +/-*^;*Arch-EGFP* ^*f/+*^, and *Slc26a5CreER* ^*T2 +/-*^;*mT/m* ^*f/+*^ mice. ABR thresholds of mice specifically expressing either ChR2-tdTomato (A) or Arch-EGFP-ER2 (B) in OHCs. ABR thresholds of mice ubiquitously expressing tdTomato without mGFP (C) or with mGFP (D) in OHCs. Values are the mean ± S.E.M.; ***: P<0.001, **: P<0.01, *: P<0.05 by two-way ANOVA followed by Student's t test with a Bonferroni correction. When ChR2-tdTomato, Arch-EGFP-ER2, mGFP and mtdTomato were heterologously expressed in OHCs (see S1 Text), knockin mice expressing either ChR2-tdTomato or mtdTomato exhibited normal hearing at all frequencies tested (4–44 kHz, A and C) while compound knockin mice expressing Arch-EGFP-ER2 exhibited normal hearing sensitivity except at 32 kHz (B) and mice expressing mGFP exhibited normal hearing sensitivity except at 22–32 kHz (D). Therefore, our subsequent FRAP analysis was performed in isolated OHCs from the apical turns of cochleae (approximately 4–16 kHz regions of cochleae) of these mice, where hearing was wild-type like *in vivo*.
(TIF)

S8 Fig Mobility of Arch-EGFP-ER2, mtdTomato, and mGFP in the lateral wall of OHCs by FRAP analysis. The OHCs used were prepared from *Slc26a5-CreER* ^*T2 +/-*^;*Arch-EGFP-ER2* ^*f/+*^ mice at P18-22. (A-D) OHCs expressing Arch-EGFP-ER2 are shown. Untreated (A), salicylate/MβCD-treated (B), salicylate-treated (C), and MβCD-treated (D) OHCs from *Slc26a5CreER* ^*T2 +/-*^;*Arch-EGFP-ER2* ^*f/+*^ mice after tamoxifen was intraperitoneally injected at P6 and P7 are shown. (E) The normalized fluorescence recovery curves for images A-D are shown. The OHCs used in this study were prepared from *Slc26a5-CreERT2* ^*+/-*^;*mT/mG* ^*f/+*^ mice at P18-22. OHCs expressing mtdTomato either without mGFP (F-I) or with mGFP (K-N) are shown. FRAP examples for untreated (F), MβCD-treated (G), salicylate-treated (H), and salicylate/MβCD-treated (I) OHCs expressing mtdTomato alone from *mT/mG* ^*f/+*^ mice are shown. (J) The normalized fluorescence recovery curves for F-I in bleached spots is shown. FRAP examples for untreated (K), MβCD-treated (L), salicylate-treated (M), and salicylate/MβCD-treated (N) OHCs from *mT/mG* ^*f/+*^ mice after tamoxifen was intraperitoneally injected at P 6–7 are shown. Initial ten data points were taken at 2.196 s intervals and the rest of 60 data points were taken at 3 seconds intervals. (O) The normalized fluorescence recovery curves for images K-N in bleached spots is shown. White arrows in A-D, F-I, and K-N show bleached spots and the black arrow in E, J, and O indicates the time of bleaching. Scale bar expresses 10 μm. Among these three membrane proteins, lateral diffusion of Arch-EGFP-ER2 and mtdTomato also showed minimal lateral diffusion and all molecules tested increased their mobility with co-treatments of salicylate and MβCD (Kruskal—Wallis, P < 0.05; A-O; Fig 7).
(TIF)

S9 Fig OHC lateral wall structure showing the borders of a single motor complex. The OHC lateral wall has three layers. The crenelated PM and the outer membrane of a membrane bound organelle called the SSC are the outer and inner layers respectively. They define the middle layer which is called the ECiS containing an orthotropically organized cytoskeletal matrix. The matrix consists of F-actin dimers that band the cell at regular (~50 nm) intervals along the length of the OHC lateral wall and, on average, have a circumferential orientation. The F-actin bands are connected to one another by spectrin. Spectrin filaments are oriented, on average, parallel to the long axis of the OHC. Spectrin is more compliant than actin contributing to the larger electrically evoked axial (as opposed to radial) movements of the OHC. A single protein filament of unknown composition links the PM to the actin. The filaments are referred to as pillars in the literature and it has been assumed that they retain a large diameter as they span the ECiS.
(TIF)

S10 Fig Examples of length measurements for OHC lateral wall after drug treatments. After FRAP experiments, Optical sections of the fluorescence images were captured. After a 3D reconstruction, the lengths and diameters in none-treated (A), MβCD-treated (B), salicylate-treated (C), and salicylate/MβCD-treated (D) Isolated OHCs were measured as shown in this figure.
(TIF)

S1 Text Supplementary Methods. (DOCX)
