# Supplementary figures and images for: Outer Hair Cell Lateral Wall Structure Constrains the Mobility of Plasma Membrane Proteins
Source: PLoS Genet. 2015 Sep 9;11(9):e1005500. doi: 10.1371/journal.pgen.1005500 (PMC4564264; doi:10.1371/journal.pgen.1005500)

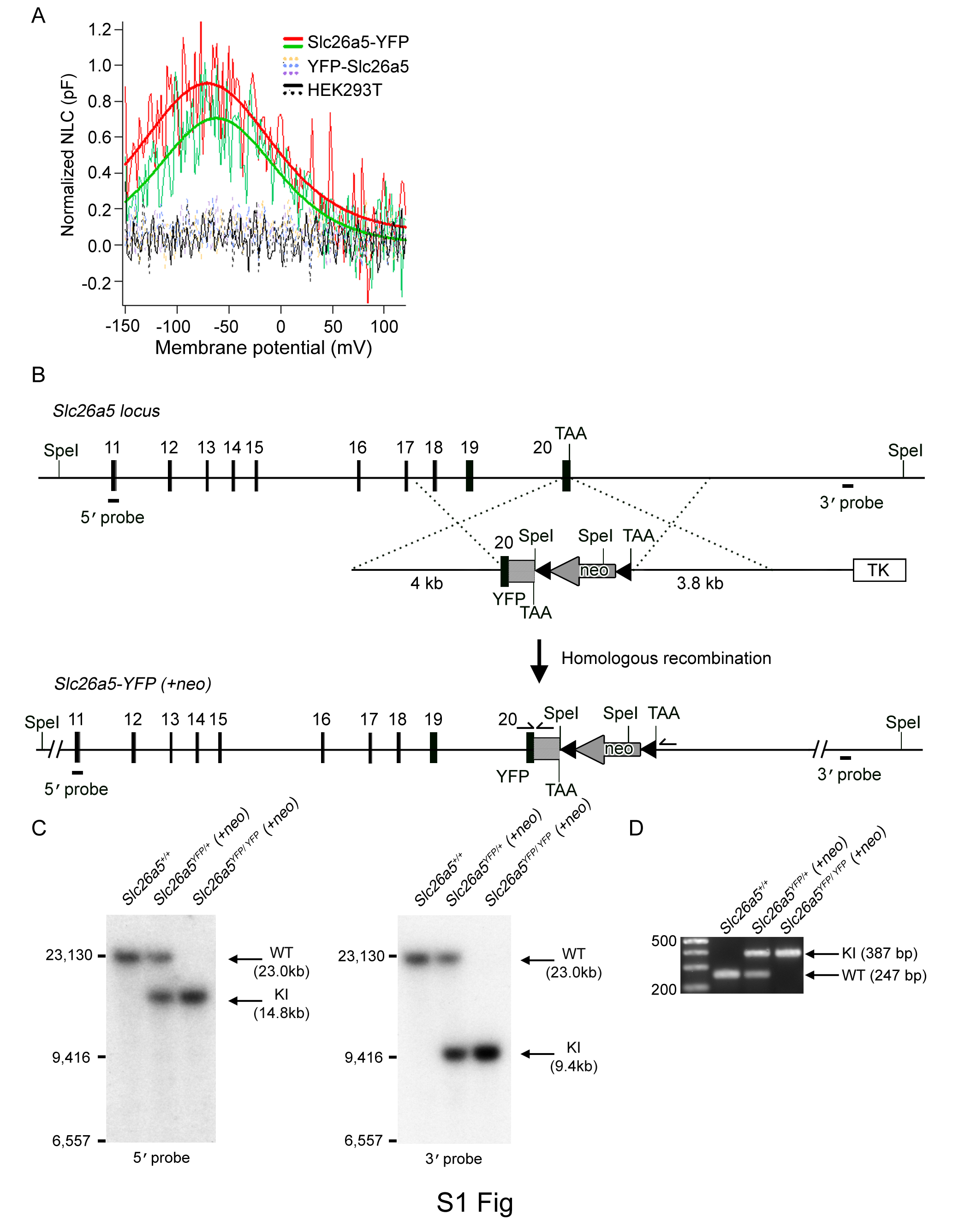

Supplement: S1 Fig — (A) NLC in Slc26a5-transfected 293T cells. YFP-Slc26a5 and Slc26a5-YFP were expressed in 293T cells, individually. NLC from Slc26a5-YFP transfected 293T cells were shown in red and green lines. YFP-Slc26a5 transfected 293T cells exhibit no NLC shown in yellow, blue, and purple dotted lines. The black line and dots show membrane capacitance at different voltages from mock transfected cells. (B) The targeted Slc26a5-YFP knockin allele. Solid rectangles represent exons 11 through 20 of Slc26a5 gene. A cassette with YFP and the neo-selectable marker flanked by loxP was inserted right before the termination codon of Slc26a5 gene. (C) Genomic southern blot analysis of Slc26a5-YFP mice. Genomic DNAs from Slc26a5 +/+, Slc26a5 YFP/+ (+neo), and Slc26a5 YFP/YFP (+neo) tails were digested with Spe I and two specific probes indicated in B were used separately. (D) PCR-based genotyping of Slc26a5 +/+, Slc26a5 YFP/+ (+neo), and Slc26a5 YFP/YFP (+neo) mice using 3 primers is indicated in B as arrows. No loss of body weight was observed in either Slc26a5 YFP/+ (+neo) or Slc26a5 YFP/YFP (+neo) mice, when compared to wild-type control. Ratio between wild-type, Slc26a5 YFP/+ (+neo), and Slc26a5 YFP/YFP (+neo) mice from heterozygous intercrosses followed approximately the Mendelian ratio. (TIF) [file pgen.1005500.s001.tif]

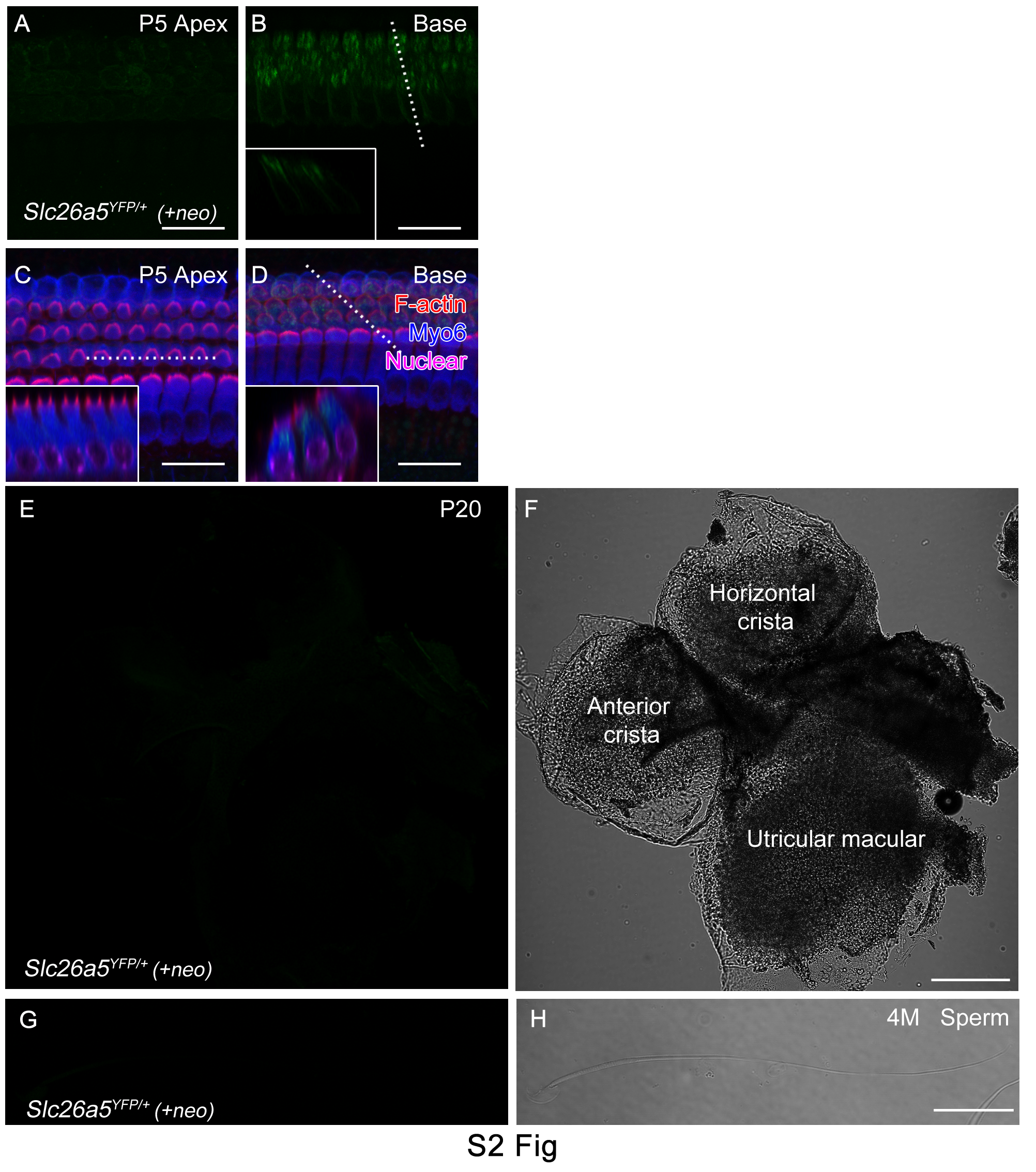

Supplement: S2 Fig — (A-D) Slc26a5-YFP fluorescence (green) in the apical (A and C) and basal (B and D) turns of a Slc26a5 YFP/+ (+neo) cochlea at P5. The dashed lines in B-D indicate the positions of optical sections shown in the insets. Myo6 (blue) was labeled as a HC marker in C and D. Enriched F-actin (red) was observed in hair bundles of OHCs in C and D. Nuclei (purple) in C and D were labeled in insets. Confocal images in A and B as well as C and D were taken with identical condition. Slc26a5-YFP fluorescence in vestibular system (E-F) and sperm (G-H) from Slc26a5 YFP/+ (+neo) mice is indicated in green. F and H shows region corresponding to E and G as differential interference contrast (DIC) images. No YFP epifluorescence were observed in vestibular system and sperm. Scale bars express 200 μm (in F), 20 μm (in A–D, and H). (TIF) [file pgen.1005500.s002.tif]

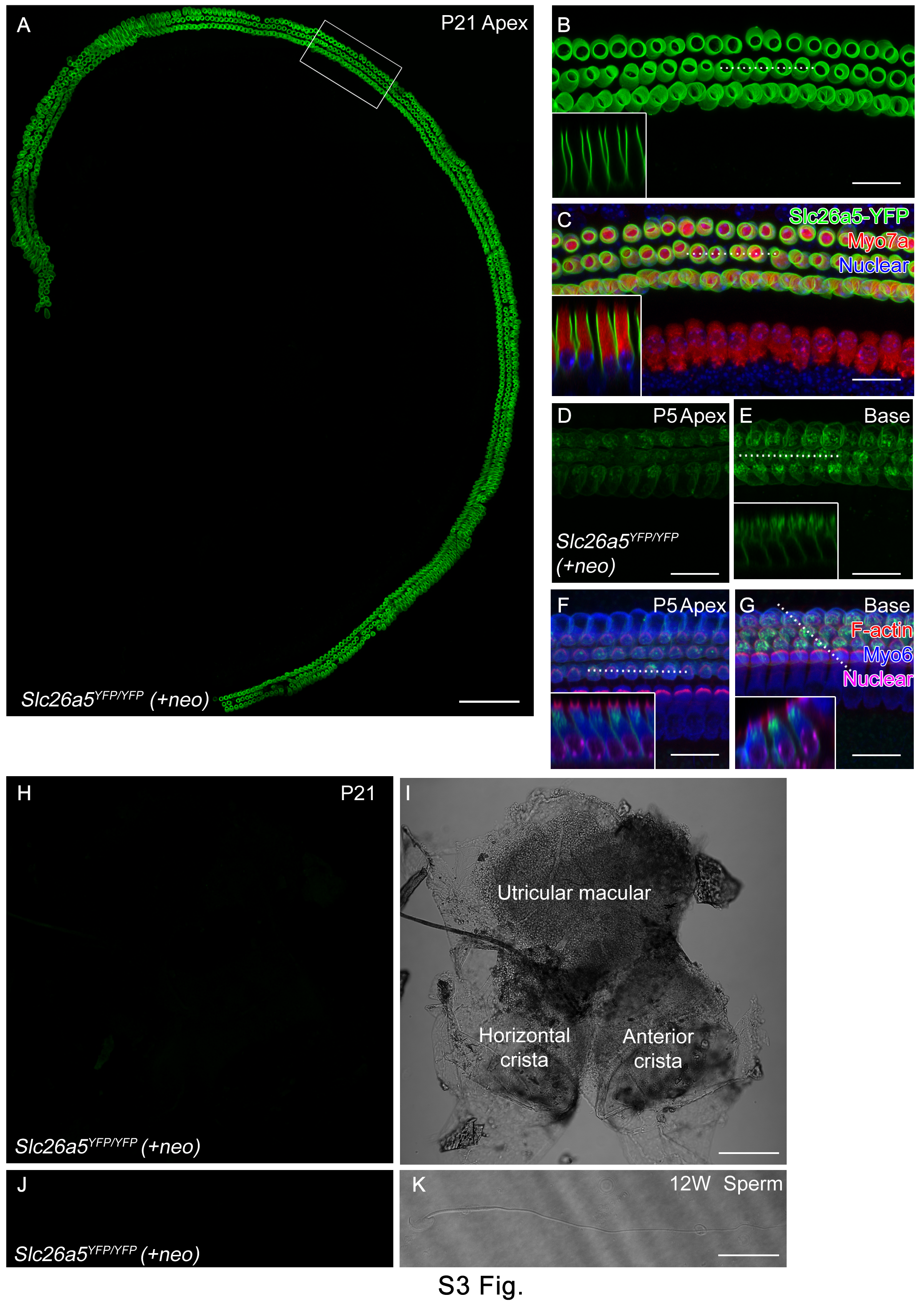

Supplement: S3 Fig — (A-J) Slc26a5-YFP distributions in Slc26a5 YFP/YFP (+neo) mice. (A-C) Slc26a5-YFP fluorescence in the apical turn of the cochleae from Slc26a5 YFP/YFP (+neo) mice at P21 are shown in green. White square in A is enlarged in B. The dashed line in B indicates the position of optical section shown in the inset. Myo7a (red) was labeled as a HC marker in C. Counter-staining of nuclei (blue) was performed using DAPI shown in C. The dashed line in C indicates the position of optical section shown in the inset. The YFP fluorescence signals were observed only in lateral wall of OHCs in cochleae. Slc26a5-YFP fluorescence in apical turn (D and F) and basal turn (E and G) of Slc26a5 YFP/YFP (+neo) cochleae at P5 are shown in green. The dashed line in E-G indicates the position of optical section shown in the inset. Myo6 was labeled as a HC marker in F-G shown in blue. Enriched F-actin (red) was observed in hair bundle of OHCs shown in F-G. Nuclei in F-G were labeled in purple in the inset. Confocal images in D and E as well as F and G were taken under identical conditions. Slc26a5-YFP fluorescence in vestibular system (H-I) and sperm (J-K) from Slc26a5 YFP/YFP (+neo) mice is indicated in green. I and K shows region corresponding to H and J as DIC images. No YFP fluorescence was observed in vestibular system and sperm. Scale bars express 200 μm (A and I), 20 μm (B-G, and K). (TIF) [file pgen.1005500.s003.tif]

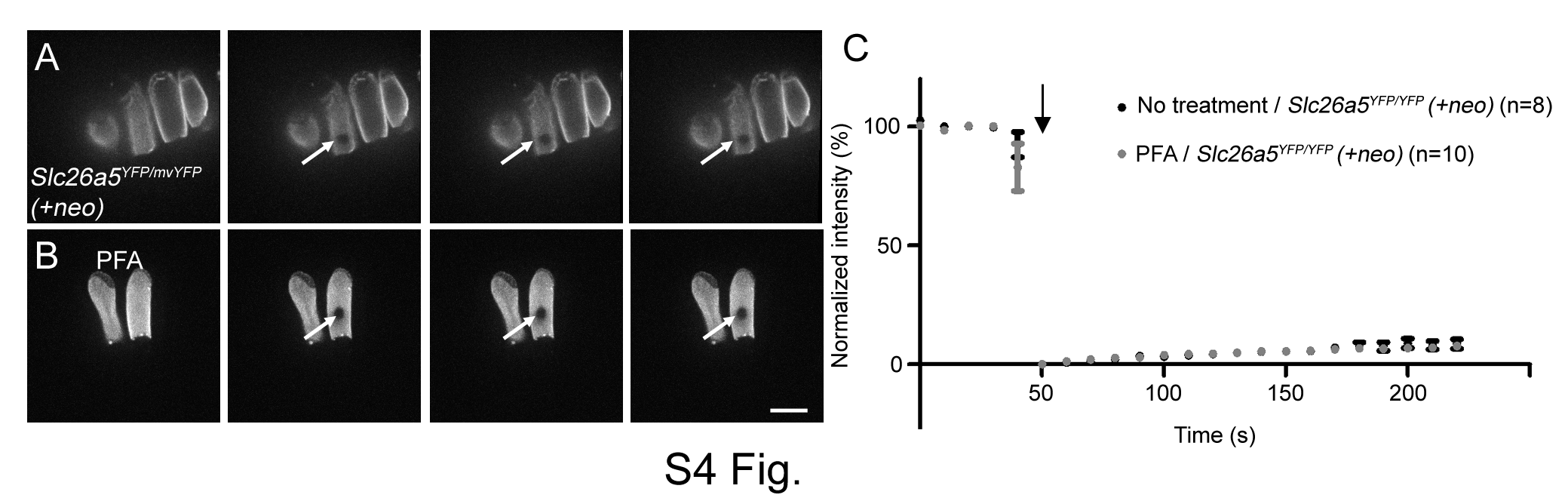

Supplement: S4 Fig — Untreated (A) and PFA-treated (B) OHCs are shown. (C) The normalized fluorescence recovery curves for Slc26a5-YFP based on fluorescence analysis of the bleached spots (see Materials and Methods). White arrows (A-B) show bleached spots and the black arrow (C) indicates the time of bleaching. Error bars express S.E.M. Scale bar expresses 10 μm. Numbers (n) of OHCs in two mice from two litters were shown. (TIF) [file pgen.1005500.s004.tif]

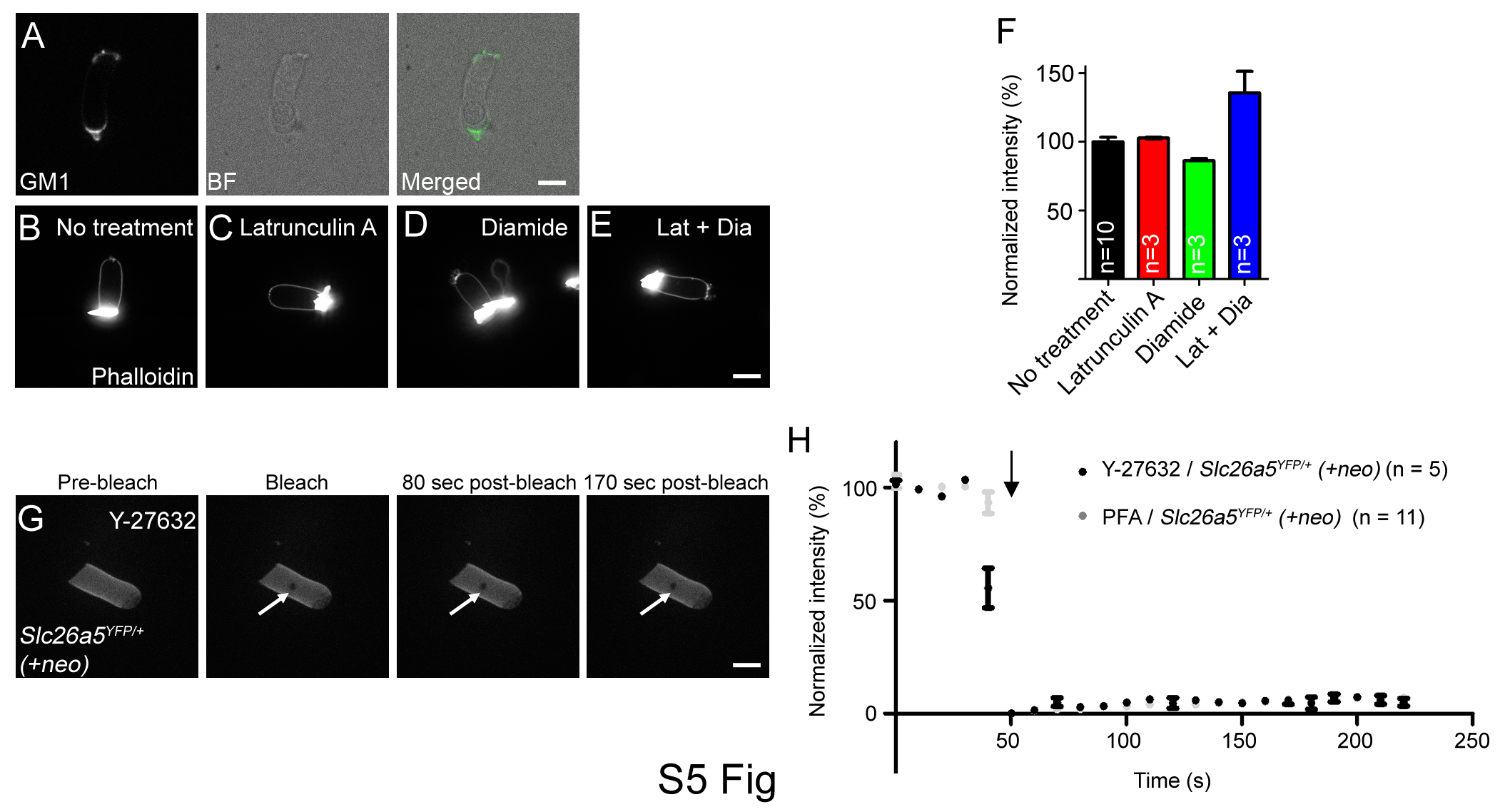

Supplement: S5 Fig — (A) GM1 distribution using Cholera Toxin Subunit B labeling experiments in lived isolated OHCs from wildtype mice at one month old of age is shown (left panel). The represented image is an optical sliced image. Image of bright field for the identical OHC is shown (middle panel). The merged image is shown in right panel. GM1 expression was below detectable range in lateral wall of OHCs. Identical results were observed from two independent mice. (B-E) F-actin distributions using Alexa Fluor 546 phalloidin labeling experiments in none-treated (B), Latrunculin A-treated (C), Diamide-treated (D), and Latrunculin A/ Diamide-treated (E) isolated OHCs from wildtype mice at one month old of age is shown (left panel). (F) Semi-quantitative analysis of Alexa Fluor 546 conjugated phalloidin’s fluorescence in OHC lateral wall from none-treated, Latrunculin A-treated, Diamide-treated, and Latrunculin A/ Diamide-treated isolated OHCs is shown. (G) FRAP examples for Y-27632-treated OHCs from Slc26a5 YFP/+ (+neo) mice at P18-22 are shown. Scale bar expresses 10 μm. (H) The normalized fluorescence recovery curves for images B are shown (see Materials and Methods). White arrows in G show bleached spots and the black arrow in H indicates the time of bleaching. Error bars express S.E.M. Numbers (n) of cells analyzed in two independent mice are shown. (TIF) [file pgen.1005500.s005.tif]

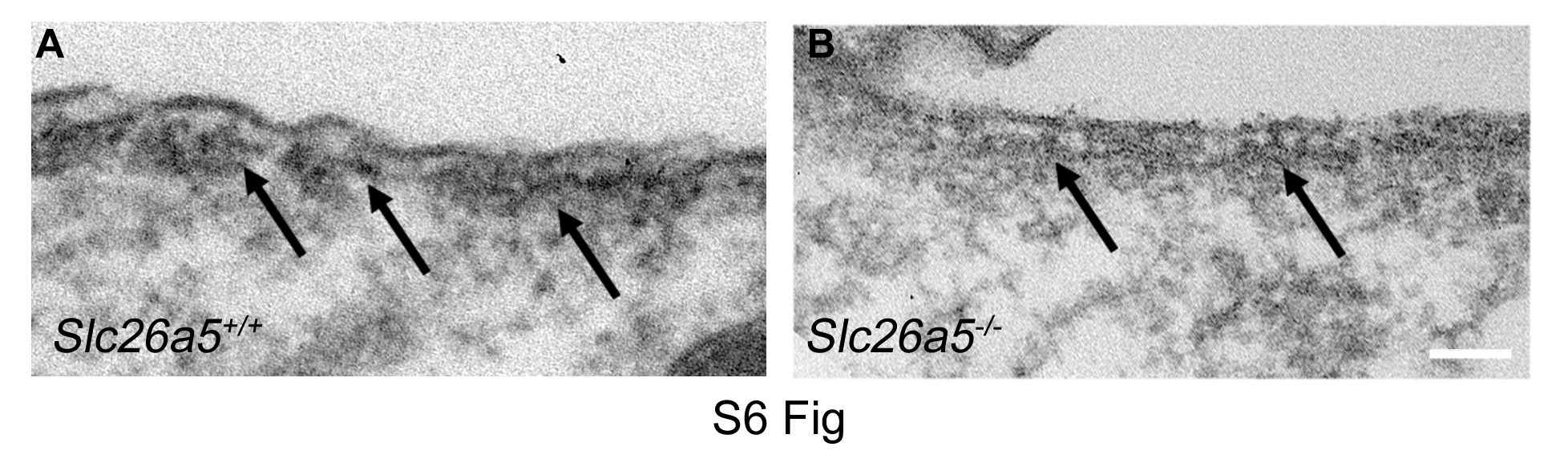

Supplement: S6 Fig — Ultrastructure of OHC lateral wall from Slc26a5 +/+ (A) and Slc26a5 -/- (B) mice are shown. Arrows indicate CL and SSC. Scale bar = 200 nm. (TIF) [file pgen.1005500.s006.tif]

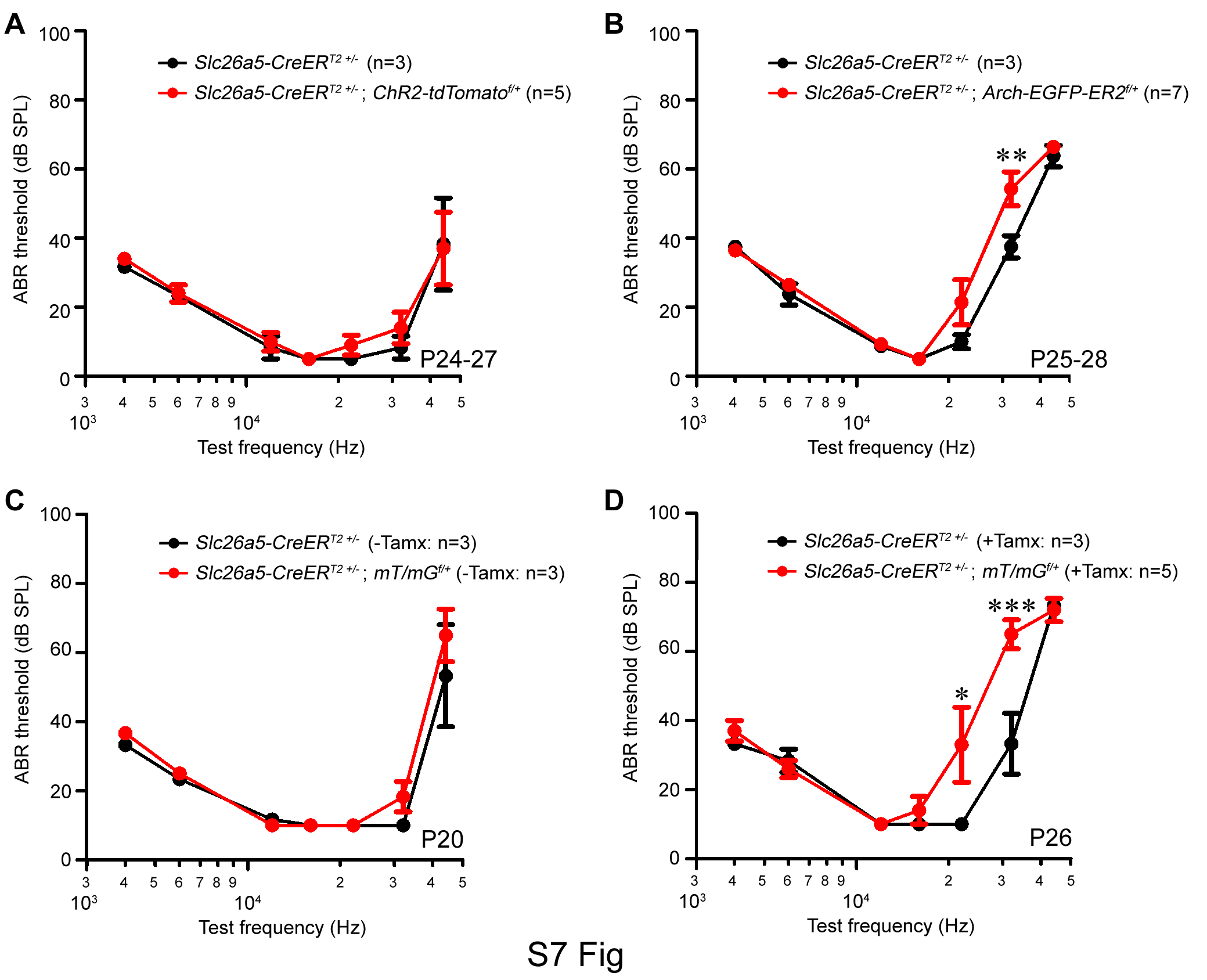

Supplement: S7 Fig — ABR thresholds of mice specifically expressing either ChR2-tdTomato (A) or Arch-EGFP-ER2 (B) in OHCs. ABR thresholds of mice ubiquitously expressing tdTomato without mGFP (C) or with mGFP (D) in OHCs. Values are the mean ± S.E.M.; ***: P<0.001, **: P<0.01, *: P<0.05 by two-way ANOVA followed by Student's t test with a Bonferroni correction. When ChR2-tdTomato, Arch-EGFP-ER2, mGFP and mtdTomato were heterologously expressed in OHCs (see S1 Text), knockin mice expressing either ChR2-tdTomato or mtdTomato exhibited normal hearing at all frequencies tested (4–44 kHz, A and C) while compound knockin mice expressing Arch-EGFP-ER2 exhibited normal hearing sensitivity except at 32 kHz (B) and mice expressing mGFP exhibited normal hearing sensitivity except at 22–32 kHz (D). Therefore, our subsequent FRAP analysis was performed in isolated OHCs from the apical turns of cochleae (approximately 4–16 kHz regions of cochleae) of these mice, where hearing was wild-type like in vivo. (TIF) [file pgen.1005500.s007.tif]

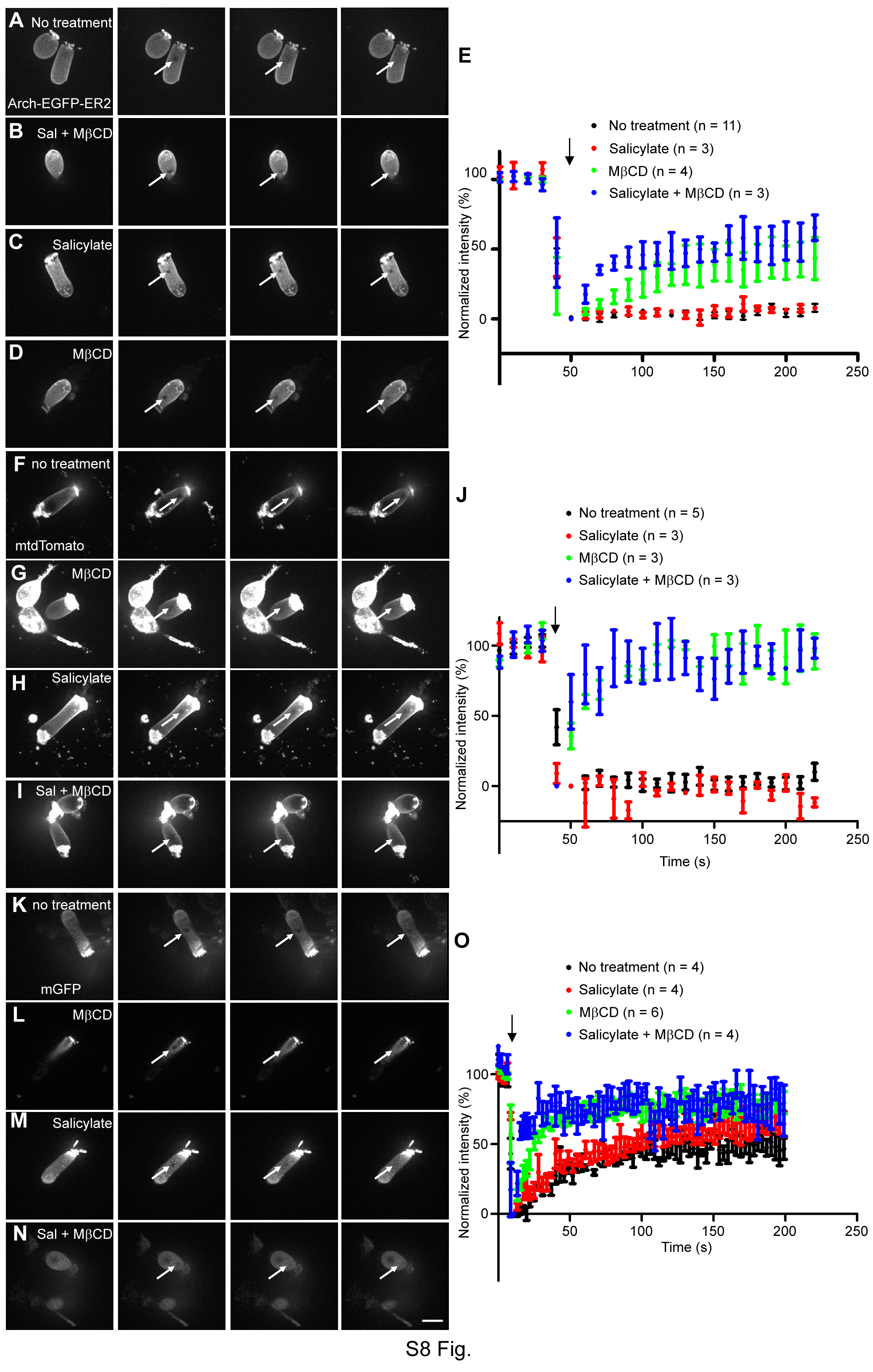

Supplement: S8 Fig — The OHCs used were prepared from Slc26a5-CreER T2 +/-;Arch-EGFP-ER2 f/+ mice at P18-22. (A-D) OHCs expressing Arch-EGFP-ER2 are shown. Untreated (A), salicylate/MβCD-treated (B), salicylate-treated (C), and MβCD-treated (D) OHCs from Slc26a5CreER T2 +/-;Arch-EGFP-ER2 f/+ mice after tamoxifen was intraperitoneally injected at P6 and P7 are shown. (E) The normalized fluorescence recovery curves for images A-D are shown. The OHCs used in this study were prepared from Slc26a5-CreERT2 +/-;mT/mG f/+ mice at P18-22. OHCs expressing mtdTomato either without mGFP (F-I) or with mGFP (K-N) are shown. FRAP examples for untreated (F), MβCD-treated (G), salicylate-treated (H), and salicylate/MβCD-treated (I) OHCs expressing mtdTomato alone from mT/mG f/+ mice are shown. (J) The normalized fluorescence recovery curves for F-I in bleached spots is shown. FRAP examples for untreated (K), MβCD-treated (L), salicylate-treated (M), and salicylate/MβCD-treated (N) OHCs from mT/mG f/+ mice after tamoxifen was intraperitoneally injected at P 6–7 are shown. Initial ten data points were taken at 2.196 s intervals and the rest of 60 data points were taken at 3 seconds intervals. (O) The normalized fluorescence recovery curves for images K-N in bleached spots is shown. White arrows in A-D, F-I, and K-N show bleached spots and the black arrow in E, J, and O indicates the time of bleaching. Scale bar expresses 10 μm. Among these three membrane proteins, lateral diffusion of Arch-EGFP-ER2 and mtdTomato also showed minimal lateral diffusion and all molecules tested increased their mobility with co-treatments of salicylate and MβCD (Kruskal—Wallis, P < 0.05; A-O; Fig 7). (TIF) [file pgen.1005500.s008.tif]

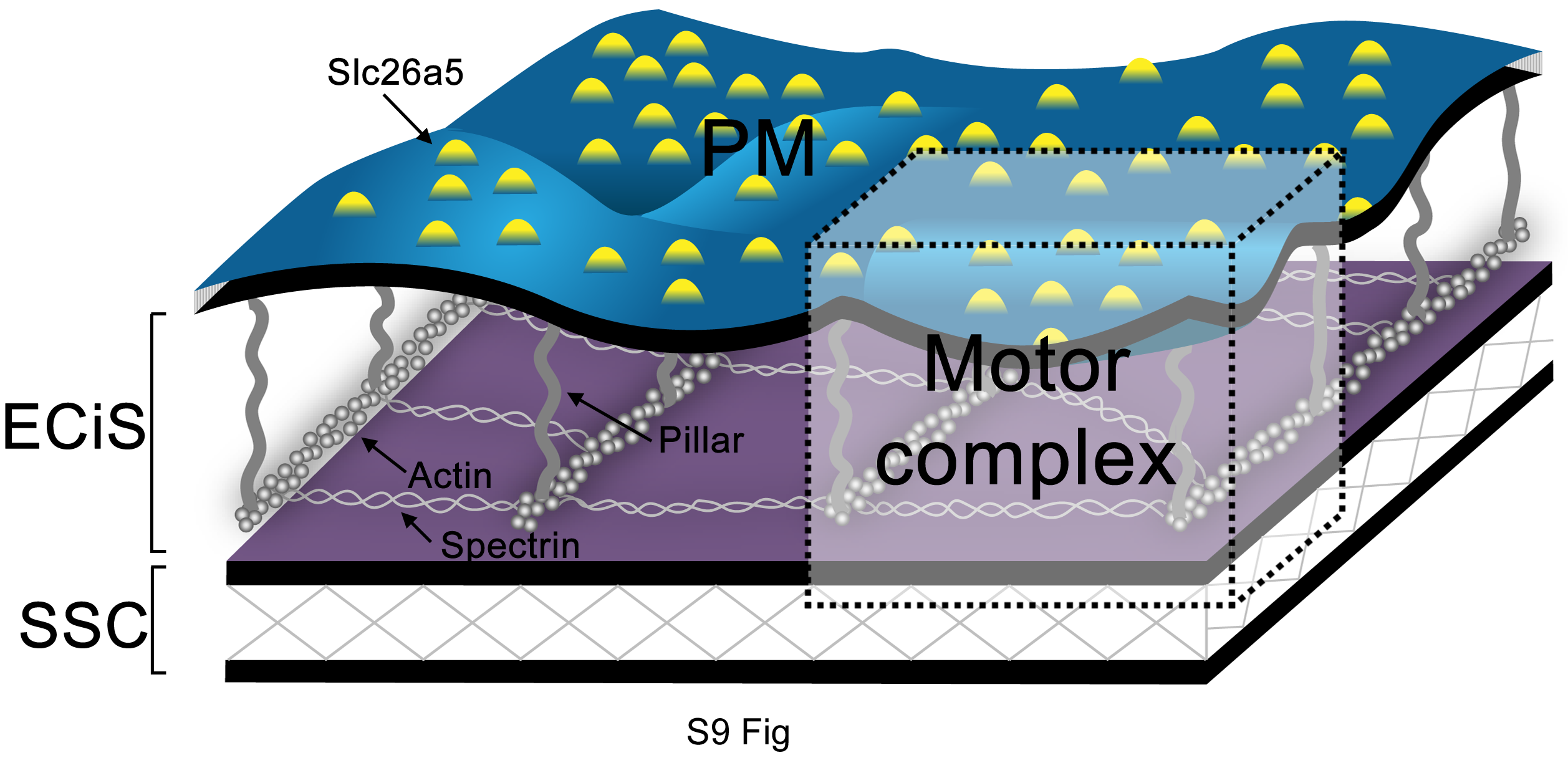

Supplement: S9 Fig — The OHC lateral wall has three layers. The crenelated PM and the outer membrane of a membrane bound organelle called the SSC are the outer and inner layers respectively. They define the middle layer which is called the ECiS containing an orthotropically organized cytoskeletal matrix. The matrix consists of F-actin dimers that band the cell at regular (~50 nm) intervals along the length of the OHC lateral wall and, on average, have a circumferential orientation. The F-actin bands are connected to one another by spectrin. Spectrin filaments are oriented, on average, parallel to the long axis of the OHC. Spectrin is more compliant than actin contributing to the larger electrically evoked axial (as opposed to radial) movements of the OHC. A single protein filament of unknown composition links the PM to the actin. The filaments are referred to as pillars in the literature and it has been assumed that they retain a large diameter as they span the ECiS. (TIF) [file pgen.1005500.s009.tif]

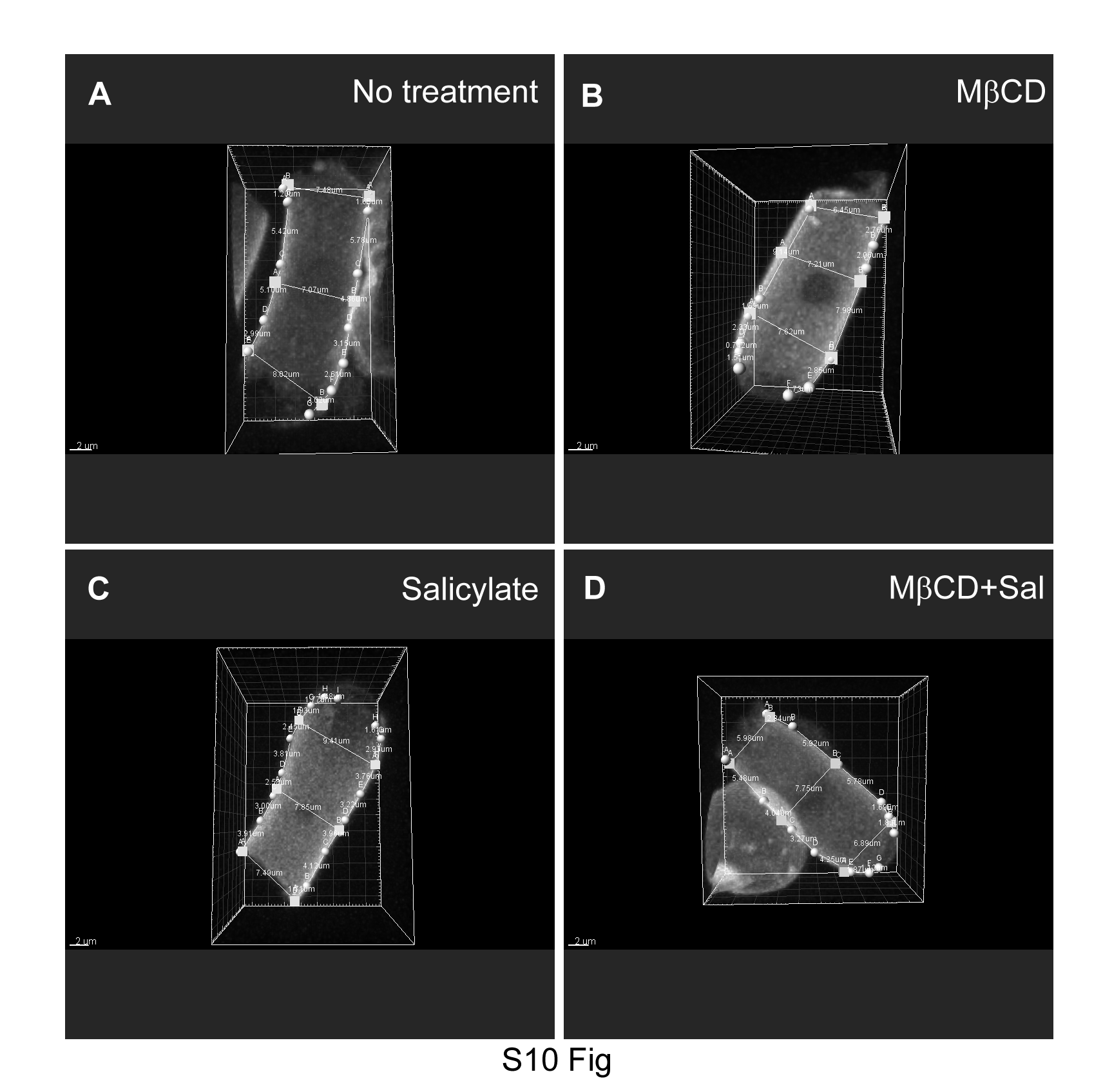

Supplement: S10 Fig — After FRAP experiments, Optical sections of the fluorescence images were captured. After a 3D reconstruction, the lengths and diameters in none-treated (A), MβCD-treated (B), salicylate-treated (C), and salicylate/MβCD-treated (D) Isolated OHCs were measured as shown in this figure. (TIF) [file pgen.1005500.s010.tif]
