# Supplementary material for: Outer Hair Cell Lateral Wall Structure Constrains the Mobility of Plasma Membrane Proteins
Source: PLoS Genet. 2015 Sep 9;11(9):e1005500. doi: 10.1371/journal.pgen.1005500 (PMC4564264; doi:10.1371/journal.pgen.1005500)
Supplement: S1 Text — (DOCX) [file pgen.1005500.s011.docx]

# Supporting information

# Supplementary Methods

## DNA transfection and nonlinear capacitance (NLC) measurements

293T cells were maintained in Dulbecco's Modified Eagle Medium (Life technologies, Gaithersburg, MD, USA) supplemented with 10% (v/v) heat-inactivated FBS, at 37°C in a humidified atmosphere containing 5% CO_2_. The cells were seeded onto 1.0 × 10^6^ cells / 35-mm dish the day before transfection. The transfection was performed using Fugene6™ (Rosch Diagnostics, Mannheim, Germany) using 1 µg of plasmid DNA. 24 hours after transfection, the transfected cells were trypsinized and the NLC was measured as described before [1].

## Gene targeting

*Slc26a5-YFP* knockin mice were generated as previously described [2]. Briefly, the targeting vector containing YFP (monomer Venus yellow fluorescent protein) was constructed (S1B Fig) and transfected into 129/SvEv ES cells (Millipore, Bedford, MA, USA). Southern blot using the homologous recombinant ES cells revealed that the homologous recombination event occurred. To create chimeric mice, the ES cells obtained were injected into the C57/BL6 blastocyst and embryos were transplanted into foster mice.

## Genotyping

The following polymerase chain reaction (PCR) primers were designed to detect the *YFP*: common primer, 5’- AGG AGG ATA TGG AGC CCA ATG CCA CAC -3’; wild-type primer, 5’- TAT AAG TGC AAG AGG CCT GTT AAT CTT TG - 3’; knockin primer, 5’- TCG TCC TTG AAG AAG ATG GTG CGC TC - 3’.

## Animals

Mice were housed under a 12 h light/dark cycle with free access to food and water. The Animal Care and Use Committees of St. Jude Children’s Research Hospital and Regional Ethics board in North Stockholm approved all of the protocols performed in this study. Genotyping of *Slc26a5-CreER^T2^* mice was previously described [3]. *FgfR3-iCreER* and Atoh1-Cre transgenic mice were kindly provided by Drs. William D Richardson and Gan Lin, respectively [4,5]. EIIA-Cre (Stock# 003314), Arch-EGFP-ER2 (Stock# 012735), eNpHR3.0-EYFP (Stock# 014539), ChR2-tdTomato (Stock# 012567), and mT/mG (Stock# 007576) mice were purchased from The Jackson Laboratory.

To delete the *neo* cassette in Slc26a5-YFP knockin mice, *Slc26a5^YFP/+^* males were crossed with the ubiquitous Cre expressing line EIIA-Cre females [6]. The germline transmission of the deleted gene was confirmed by PCR analysis as previously described [3].

In order to ectopically express Arch-EGFP-ER2, Channelrhodopsin-2(H134R)–tdTomato (ChR2-tdTomato), membrane-bound GFP (mGFP) or membrane-bound tdTomato (mtdTomato) in OHCs, *Slc26a5-CreER^T2^* homozygous mice were crossed with *Arch-EGFP-ER2^f/+^*, *ChR2-tdTomato^f/+^*, or *mT/mG^f/+^* mice. *Slc26a5-CreER^T2^* mice containing *IRES-CreER^T2^* following the Slc26a5 termination codon exhibited induced Cre activities specifically in OHCs after intraperitoneal injection of tamoxifen [3]. Moreover, each channelrhodopsin gene was inserted behind the *floxed stop cassette* that was driven by the CAG promoter at *Rosa26 locus* in *Arch-EGFP-ER2^f/+^* and *ChR2-tdTomato^f/+^* mice. Therefore, deletion of the *floxed cassette* by Cre activities induces the channelrhodopsins in Cre+ cells in mice. The *mT/mG* mice possess *floxed mtdTomato* coding sequence followed by *mGFP* coding sequence on the *Rosa26 locus* and the expression is driven by CMV β-actin enhancer-promoter, which achieves ubiquitous expression [7]. Therefore, mtdTomato is expressed ubiquitously and the deletion of the *floxed mtdTomato cassette* by Cre activities induces the mGFP expression instead in Cre+ cells.

To investigate ChR2-tdTomato mobility in Deiters’ cells, *FgfR3-iCreER^T2^* transgenic mice were crossed with *ChR2-tdTomato^f/+^* mice. The induced Cre activities in *FgfR3-iCreER^T2^* transgenic mice were observed in Deiters’ cells and pillar cells when tamoxifen was intraperitoneally injected once a day at P6 and P7 [8]. Deiters’ cells overexpressing ChR2-tdTomato were identified by its shape (see Fig. 7H). Administration of tamoxifen to induce Cre recombination *in vivo* was described previously [9].

Creation of Slc26a5 knockout mice has been described before [10]. To heterologously express ChR2-tdTomato in *Slc26a5^-/-^* hair cells, *Slc26a5^+/-^;tdTomato^f/+^* mice were crossed with Slc26a5^+/-^; Atoh1-Cre^+^ mice.

## Histological analysis

Cochlear whole mount prepared as previously described [1]. Primary antibodies used were goat anti-Slc26a5 antibody (N-20; 1:200 dilution, Santa Cruz Biotechnology, Santa Cruz, CA, USA), rabbit anti-myo-7a antibody (1:100 dilution, Proteus Bioscience), rabbit anti-myo6 antibody conjugated to Alexa647 (1:100 dilution, Proteus Bioscience), and Alexa Fluor 546 phalloidin (1:100 dilution, Invitrogen, Carlsbad, CA). The immunofluorescence was visualized by adding Alexa Fluor 488 chicken anti-goat IgG (H+L) and Alexa Fluor 596 chicken anti-rabbit IgG (H+L) (Molecular Probes, Eugene, OR, USA). Counter-staining of nuclei was performed using 4′,6-diamidino-2-phenylindole (DAPI, SIGMA, St-Louis, MO, USA). Fluorescence images were analyzed with a Zeiss Axiophot2 microscope equipped with a 40× oil immersion and 1.4 NA objective and were captured at 0.6 µm intervals from the upper to lower edges by using a LSM700 confocal laser scanning image system (Carl Zeiss, Jena, Germany). Represented images were obtained by expanding the tonal range of the images adjusting white and black points using Photoshop CS2 (Adobe Systems, San Jose, CA, USA)

## F-actin staining

Dissociated cells from cochleae were placed on poly-L-lysin coated cover slips and stained with Alexa Fluor® 546 phalloidin (Life technology). The concentration used was 1:100.

## Auditory brainstem response (ABR) measurement

Measurement of hearing sensitivities using opened field ABR was described previously [1]. Briefly, ABR waveforms were recorded in a sound booth (Industrial Acoustic Company, Bronx, NY) using subdermal needles positioned in the skull, below the pinna and at the base of the tail at 5 cm away from a Multi-Field (MF1) magnetic speaker (Tucker-Davis Technologies (TDT)) and the responses were fed into low-impedance Medusa Digital Biological Amplifier System (RA4L, TDT; 20 dB gain). At each frequency, the stimulus intensity was reduced from 75 to 0 dB in 5 dB steps to determine the threshold dB SPL. The recorded signals were filtered by a band-pass filter from 300 Hz to 3 kHz and ABR waveforms were averaged in response to 500 tone bursts. Calibrated tone bursts (200 kHz sampling rate) using PCB 377C10 microphone (PCB Piezotronics, Inc. New York, NY) were produced using a BioSigRZ system (TDT, Alachua, FL, RZ6,) and delivered through a MF1 magnetic speaker at a rate of 21/s at frequencies of 4, 6, 12, 16, 22, 32 and 44 kHz.

## Measurement of NLC from isolated outer hair cells

Cochleae were harvested from mice of different genotypes at P20-P26. The organs of Corti in approximately 4 to 16 kHz regions were taken out and digested with 1 mg/mL collagenase type IV (SIGMA) for 3-5 min. The enzyme digested-OHCs were triturating gently three times using a fire-polished plastic pipette. The isolated OHCs were bathed in external solution. The external solution was 120 mM NaCl, 20 mM TEA-Cl, 2 mM CoCl_2_, 2 mM MgCl_2_, 10 mM HEPES. The osmolarity and pH were adjusted to 305-310 mOsm/kg and 7.3. Healthy cylindrical OHCs were selected. When either any Brownian motions inside of OHCs or swollen isolated OHCs were observed in the course of experiments, the data was discarded. All of data was collected at room temperature within two hours after sacrificing mice.

Borosilicate glass capillaries with inner filaments (1B150F-4, World Precision Instruments, Sarasota, FL, USA) were pulled using a pipette puller (P2000, Sutter Instruments, Novato, CA, USA) and fire-polished to obtain patch pipette. The obtained patch pipette was filled using intracellular solution. The internal solution was 140 mM CsCl, 2 mM CaCl_2_, 5 mM EGTA, Cs-HEPES (pH 7.3). The resistance of the pipettes was 3.5 to 5.5 MΩ. When tight seal (Giga seal) was achieved between the pipettes and OHC membrane, the pipette capacitance was compensated manually (Axon patch 200B instrument, Molecular Devices, Sunnyvale, CA, USA). The whole-cell configuration was achieved by rupturing the OHC membrane under the pipette tip. Current responses were measured using jClamp (SciSoft, CT) by a two-sine voltage stimulus protocol (10 mV peak at both 390.6 and 781.2 Hz [11]). These small sinusoidal voltage stimuli were superimposed onto voltage sinusoidal stimulus that spanned ±150 mV. NLC was obtained by fast Fourier transform-based admittance analysis [12]. Voltage stimulus errors due to series resistances were corrected after data collection. The obtained NLC data were fitted to a derivative of the two-state Boltzmann function that involves linear capacitance *C*_lin_, maximum nonlinear charge transfer *Q*_max_, the voltage at peak capacitance *V*_pk_, and slope factor α (Eq. 1). The curve fitting was performed using the Igor Pro 6.1.2.1 (WaveMetrics, Lake Owego, OR, USA).

$C_{m}(V)=C_{lin}+\frac{Q_{\max}}{\alpha e^{\frac{V-V_{pk}}{\alpha}}(1+e^{-\frac{V-V_{pk}}{\alpha}})^{2}}$ Eq. 1

## Min6m9 cell culture

Min6m9 cells were cultured in Dulbecco's Modified Eagle Medium (DMEM; Life Technologies) containing 11.1 mM glucose and supplemented with 100 U/ml penicillin, 100 μg/ml streptomycin, 2 mM glutamine, 10% heat-inactivated fetal calf serum, and 75 μM β-mercaptoethanol at 37°C in a humidified atmosphere containing 5% CO_2_. Min6m9 cells were seeded onto 35mm culture dishes and transfected with pRc.CMi.mVenus (plasmid expressing YFP under the control of the CMV promoter) using lipofectamine (Life Technologies), and cultured further for 24 hours in the culture medium described above.

## Sample preparation for transmission electron microscopy

Apical turns of cochleae corresponding to approximately 4 to 16 kHz regions were harvested from mice of different genotypes at P20-P24. The whole mounts were quickly fixed (within one minute) using a PELCO Biowave Pro microwave tissue processor equipped with a Pelco ColdSpot Pro (Ted Pella, Redding, CA, USA) at 150W and 20’’ Hg in 2.5% glutaraldehyde in phosphate-buffer saline (PBS) and immediately recovered in 0.01M glycine in PBS to reduce possible artifacts due to chemical cross-linking. Samples were then high-pressure frozen using 20% glycerol as a cryoprotectant (Bal-Tec, Los Angeles, CA, USA) and freeze substituted with 1% osmium tetroxide, 0.1% uranyl acetate, and 5% double-distilled water [[68](#_ENREF_68)]. The dehydrated tissue was subsequently embedded into epon resin and 70-120 nm thick sections were collected as described in Materials and Methods.

# Supplementary references

1. Yamashita T, Fang J, Gao J, Yu Y, Lagarde MM, et al. (2012) Normal hearing sensitivity at low-to-middle frequencies with 34% prestin-charge density. PLoS One 7: e45453.

2. Gao J, Wang X, Wu X, Aguinaga S, Huynh K, et al. (2007) Prestin-based outer hair cell electromotility in knockin mice does not appear to adjust the operating point of a cilia-based amplifier. Proc Natl Acad Sci U S A 104: 12542-12547.

3. Fang J, Zhang WC, Yamashita T, Gao J, Zhu MS, et al. (2012) Outer hair cell-specific prestin-CreERT2 knockin mouse lines. Genesis 50: 124-131.

4. Yang H, Xie X, Deng M, Chen X, Gan L (2010) Generation and characterization of Atoh1-Cre knock-in mouse line. Genesis 48: 407-413.

5. Young KM, Mitsumori T, Pringle N, Grist M, Kessaris N, et al. (2010) An Fgfr3-iCreER(T2) transgenic mouse line for studies of neural stem cells and astrocytes. Glia 58: 943-953.

6. Lakso M, Pichel JG, Gorman JR, Sauer B, Okamoto Y, et al. (1996) Efficient in vivo manipulation of mouse genomic sequences at the zygote stage. Proc Natl Acad Sci U S A 93: 5860-5865.

7. Muzumdar MD, Tasic B, Miyamichi K, Li L, Luo L (2007) A global double-fluorescent Cre reporter mouse. Genesis 45: 593-605.

8. Cox BC, Liu Z, Lagarde MM, Zuo J (2012) Conditional gene expression in the mouse inner ear using Cre-loxP. J Assoc Res Otolaryngol 13: 295-322.

9. Chow LM, Tian Y, Weber T, Corbett M, Zuo J, et al. (2006) Inducible Cre recombinase activity in mouse cerebellar granule cell precursors and inner ear hair cells. Dev Dyn 235: 2991-2998.

10. Liberman MC, Gao J, He DZ, Wu X, Jia S, et al. (2002) Prestin is required for electromotility of the outer hair cell and for the cochlear amplifier. Nature 419: 300-304.

11. Santos-Sacchi J, Kakehata S, Takahashi S (1998) Effects of membrane potential on the voltage dependence of motility-related charge in outer hair cells of the guinea-pig. J Physiol 510 ( Pt 1): 225-235.

12. He DZ, Jia S, Sato T, Zuo J, Andrade LR, et al. (2010) Changes in plasma membrane structure and electromotile properties in prestin deficient outer hair cells. Cytoskeleton (Hoboken) 67: 43-55.

# Supplementary figure legends:

## S1 Fig. Slc26a5-YFP knockin mouse strategy

(A) NLC in Slc26a5-transfected 293T cells. YFP-Slc26a5 and Slc26a5-YFP were expressed in 293T cells, individually. NLC from Slc26a5-YFP transfected 293T cells were shown in red and green lines. YFP-Slc26a5 transfected 293T cells exhibit no NLC shown in yellow, blue, and purple dotted lines. The black line and dots show membrane capacitance at different voltages from mock transfected cells. (B) The targeted Slc26a5-YFP knockin allele. Solid rectangles represent exons 11 through 20 of *Slc26a5* gene. A cassette with *YFP* and the *neo*-selectable marker flanked by loxP was inserted right before the termination codon of *Slc26a5* gene. (C) Genomic southern blot analysis of Slc26a5-YFP mice. Genomic DNAs from *Slc26a5^+/+^*, *Slc26a5^YFP/+^*, and *Slc26a5^YFP/YFP^* tails were digested with Spe I and two specific probes indicated in B were used separately. (D) PCR-based genotyping of *Slc26a5^+/+^*, *Slc26a5^YFP/+^*, and *Slc26a5^YFP/YFP^* mice using 3 primers is indicated in B as arrows. No loss of body weight was observed in either *Slc26a5^YFP/+^* or *Slc26a5^YFP/YFP^* mice, when compared to wild-type control. Ratio between wild-type, *Slc26a5^YFP/+^*, and *Slc26a5^YFP/YFP^* mice from heterozygous intercrosses followed approximately the Mendelian ratio.

## S2 Fig. Slc26a5-YFP recapitulates endogenous Slc26a5 distribution in *Slc26a5^YFP/+^ (+neo)* mice at neonatal stages.

(A-D) Slc26a5-YFP fluorescence (green) in the apical (A and C) and basal (B and D) turns of a *Slc26a5^YFP/+^* *(+neo)* cochlea at P5. The dashed lines in B-D indicate the positions of optical sections shown in the insets. Myo6 (blue) was labeled as a HC marker in C and D. Enriched F-actin (red) is observed in hair bundles of OHCs in C and D. Nuclei (purple) in C and D are labeled in insets. Confocal images in A and B as well as C and D were taken with identical condition. Slc26a5-YFP fluorescence in vestibular system (E-F) and sperm (G-H) from *Slc26a5^YFP/+^ (+neo)* mice is indicated in green. F and H shows region corresponding to E and G as differential interference contrast (DIC) images. No YFP epifluorescence were observed in vestibular system and sperm. Scale bars express 200 μm (in F), 20 μm (in A–D, and H).

## S3 Fig. Slc26a5-YFP recapitulates endogenous Slc26a5 distribution and is functional in *Slc26a5^YFP/YFP^ (+neo)* mice.

(A-J) Slc26a5-YFP distributions in *Slc26a5^YFP/YFP^* *(+neo)* mice. (A-C) Slc26a5-YFP fluorescence in the apical turn of the cochleae from *Slc26a5^YFP/YFP^ (+neo)* mice at P21 are shown in green. White square in A is enlarged in B. The dashed line in B indicates the position of optical section shown in the inset. Myo7a (red) was labeled as a HC marker in C. Counter-staining of nuclei (blue) was performed using DAPI shown in C. The dashed line in C indicates the position of optical section shown in the inset. The YFP fluorescence signals were observed only in lateral wall of OHCs in cochleae. Slc26a5-YFP fluorescence in apical turn (D and F) and basal turn (E and G) of *Slc26a5^YFP/YFP^ (+neo)* cochleae at P5 are shown in green. The dashed line in E-G indicates the position of optical section shown in the inset. Myo6 was labeled as a HC marker in F-G shown in blue. Enriched F-actin (red) is observed in hair bundle of OHCs shown in F-G. Nuclei in F-G are labeled in purple in the inset. Confocal images in D and E as well as F and G were taken under identical conditions. Slc26a5-YFP fluorescence in vestibular system (H-I) and sperm (J-K) from *Slc26a5^YFP/YFP^ (+neo)* mice is indicated in green. I and K shows region corresponding to H and J as DIC images. No YFP fluorescence was observed in vestibular system and sperm. Scale bars express 200 μm (A and I), 20 μm (B-G, and K).

## S4 Fig. Slc26a5 exhibits minimal lateral mobility in the lateral wall of isolated OHCs from *Slc26a5^YFP/YFP^ (+neo)* mice at P18-22 using FRAP analysis.

## Untreated (A) and PFA-treated (B) OHCs are shown. (C) The normalized fluorescence recovery curves for Slc26a5-YFP based on fluorescence analysis of the bleached spots (see Materials and Methods). White arrows (A-B) show bleached spots and the black arrow (C) indicates the time of bleaching. Error bars express S.E.M. Scale bar expresses 10 μm. Numbers (n) of OHCs in two mice from two litters were shown.

## S5 Fig. GM1 and F-actin distribtuions and Slc26a5 mobility analysis in the lateral wall of isolated OHCs after treatment with Y-27632.

(A) GM1 distribution using Cholera Toxin Subunit B labeling experiments in lived isolated OHCs from wildtype mice at one month old of age is shown (left panel). The represented image is an optical sliced image. Image of bright field for the identical OHC is shown (middle panel). The merged image is shown in right panel. GM1 expression was below detectable range in lateral wall of OHCs. Identical results were observed from two independent mice. (B-E) F-actin distributions using Alexa Fluor 546 phalloidin labeling experiments in none-treated (B), Latrunculin A-treated (C), Diamide-treated (D), and Latrunculin A/ Diamide-treated (E) isolated OHCs from wildtype mice at one month old of age is shown (left panel). (F) Semi-quantitative analysis of Alexa Fluor 546 conjugated phalloidin’s fluorescence in OHC lateral wall from none-treated, Latrunculin A-treated, Diamide-treated, and Latrunculin A/ Diamide-treated isolated OHCs is shown. (G) FRAP examples for Y-27632-treated OHCs from *Slc26a5^YFP/+^* *(+neo)* mice at P18-22 are shown. Scale bar expresses 10 μm. (H) The normalized fluorescence recovery curves for images B are shown (see Materials and Methods). White arrows in G show bleached spots and the black arrow in H indicates the time of bleaching. Error bars express S.E.M. Numbers (n) of cells analyzed in two independent mice are shown.

## **S6 Fig.** The SSC is present in OHCs from *Slc26a5^-/-^* mice.

## Ultrastructure of OHC lateral wall from *Slc26a5^+/+^* (A) and *Slc26a5^-/-^* (B) mice are shown. Arrows indicate CL and SSC. Scale bar = 200 nm.

## S7 Fig. ABR thresholds of *Slc26a5CreER^T2 +/-^;ChR2-tdTomato^f/+^*, *Slc26a5CreER^T2 +/-^;Arch-EGFP^f/+^*, and *Slc26a5CreER^T2 +/-^;mT/m^f/+^* mice.

Hearing sensitivity of compound transgenic mice. ABR thresholds of mice specifically expressing either ChR2-tdTomato (A) or Arch-EGFP-ER2 (B) in OHCs. ABR thresholds of mice ubiquitously expressing tdTomato without mGFP (C) or with mGFP (D) in OHCs. Values are the mean ± S.E.M.; ***: P<0.001, **: P<0.01, *: P<0.05 by two-way ANOVA followed by Student's t test with a Bonferroni correction. When ChR2-tdTomato, Arch-EGFP-ER2, mGFP and mtdTomato were heterologously expressed in OHCs (see Supplementary Methods), knockin mice expressing either ChR2-tdTomato or mtdTomato exhibited normal hearing at all frequencies tested (4-44 kHz, A and C) while compound knockin mice expressing Arch-EGFP-ER2 exhibited normal hearing sensitivity except at 32 kHz (B) and mice expressing mGFP exhibited normal hearing sensitivity except at 22-32 kHz (D). Therefore, our subsequent FRAP analysis was performed in isolated OHCs from the apical turns of cochleae (approximately 4-16 kHz regions of cochleae) of these mice, where hearing was wild-type like *in vivo*.

## S8 Fig. Mobility of Arch-EGFP-ER2, mtdTomato, and mGFP in the lateral wall of OHCs by FRAP analysis.

The OHCs used were prepared from *Slc26a5-CreER^T2 +/-^;Arch-EGFP-ER2^f/+^* mice at P18-22. (A-D) OHCs expressing Arch-EGFP-ER2 are shown. Untreated (A), salicylate/MβCD-treated (B), salicylate-treated (C), and MβCD-treated (D) OHCs from *Slc26a5CreER^T2 +/-^;Arch-EGFP-ER2^f/+^* mice after tamoxifen was intraperitoneally injected at P6 and P7 are shown. (E) The normalized fluorescence recovery curves for images A-D are shown. The OHCs used in this study were prepared from *Slc26a5-CreERT2^+/-^;mT/mG^f/+^* mice at P18-22. OHCs expressing mtdTomato either without mGFP (F-I) or with mGFP (K-N) are shown. FRAP examples for untreated (F), MβCD-treated (G), salicylate-treated (H), and salicylate/MβCD-treated (I) OHCs expressing mtdTomato alone from *mT/mG^f/+^* mice are shown. (J) The normalized fluorescence recovery curves for F-I in bleached spots is shown. Initial ten data points were taken at 2.196 s intervals and the rest of 60 data points were taken at 3 seconds intervals. FRAP examples for untreated (K), MβCD-treated (L), salicylate-treated (M), and salicylate/MβCD-treated (N) OHCs from *mT/mG^f/+^* mice after tamoxifen was intraperitoneally injected at P 6-7 are shown. Initial ten data points were taken at 2.196 s intervals and the rest of 60 data points were taken at 3 seconds intervals. (O) The normalized fluorescence recovery curves for images K-N in bleached spots is shown. White arrows in A-D, F-I, and K-N show bleached spots and the black arrow in E, J, and O indicates the time of bleaching. Scale bar expresses 10 µm.

Among these three membrane proteins, lateral diffusion of Arch-EGFP-ER2 and mtdTomato also showed minimal lateral diffusion and all molecules tested increased their mobility with co-treatments of salicylate and MβCD (Kruskal–Wallis, P < 0.05; A-O; Table 1).

## S9 Fig. OHC lateral wall structure showing the borders of a single motor complex.

## The OHC lateral wall has three layers. The crenelated PM and the outer membrane of a membrane bound organelle called the SSC are the outer and inner layers respectively. They define the middle layer which is called the ECiS containing an orthotropically organized cytoskeletal matrix. The matrix consists of F-actin dimers that band the cell at regular (~50 nm) intervals along the length of the OHC lateral wall and, on average, have a circumferential orientation. The F-actin bands are connected to one another by spectrin. Spectrin filaments are oriented, on average, parallel to the long axis of the OHC. Spectrin is more compliant than actin contributing to the larger electrically evoked axial (as opposed to radial) movements of the OHC. A single protein filament of unknown composition links the PM to the actin. The filaments are referred to as pillars in the literature and it has been assumed that they retain a large diameter as they span the ECiS.

## S10 Fig. Examples of length measurements for OHC lateral wall after drug treatments.

## After FRAP experiments, Optical sections of the fluorescence images were captured. After a 3D reconstruction, the lengths and diameters in none-treated (A), MβCD-treated (B), salicylate-treated (C), and salicylate/MβCD-treated (D) Isolated OHCs were measured as shown in this figure.
